# The impact of dropouts in scRNAseq dense neighborhood analysis^☆^

**DOI:** 10.1016/j.csbj.2025.03.033

**Published:** 2025-03-24

**Authors:** Alisa Pavel, Manja Gersholm Grønberg, Line H. Clemmensen

**Affiliations:** a Department of Applied Mathematics and Computer Science, Technical University of Denmark, 2800, Kongens Lyngby, Denmark; b Department of Mathematical Sciences, University of Copenhagen, 2100, Copenhagen, Denmark

**Keywords:** scRNAseq, Dropouts, Clustering, Sparsity

## Abstract

Single cell RNA sequencing (scRNAseq) provides the possibility to investigate transcriptomic profiles on a single cell level. However, the data show unique challenges in comparison to bulk transcriptomic data, one being high dropout rates, which yields high sparsity data. Many classical analysis and preprocessing pipelines are based on the assumption that poor data can be counteracted by quantity and that similar cells (samples) are close to each other in space. Clustering is commonly used to detect clusters (dense local cell neighborhoods) under the assumption that similar cells are close to each other in space (where close is dependent on the (distance) metric used). The most commonly used clustering methodologies to detect dense local neighborhoods are based on graph clustering on a nearest neighbor graph. However, high dropout rates may break this assumption and make it difficult to reliably detect such dense local neighborhoods.

We assess the cluster homogeneity and stability under increasing degrees of dropouts in one of the most popular clustering pipelines (dimensionality reduction + graph based clustering), as provided by scRNAseq analyses packages Seurat and Scanpy. Our study showcases that while the default pipeline performs well in terms of cluster homogeneity (i.e., cells in a cluster are of the same type), also with increasing dropout rates, the stability of clusters (i.e., cell pairs consistently being in the same cluster) decreases. This implies that sub-populations within cell types are increasingly difficult to identify under increasing dropout rates because observations are not consistently close.

Our results challenge the current practice of using default clustering pipelines and the general assumption of identifiable local neighborhoods on high dropout data. Hence, these results suggest that careful consideration in interpretation and downstream analysis need to be made when relying on local neighborhoods and clusters on scRNAseq data. In addition, these results call for extensive benchmarking, to identify and provide methods robust in their local neighborhood relationships on data containing low to high dropout rates.

## Highlights

• We investigate the assumptions under which scRNAseq uses clustering to identify local neighborhoods of similar cells.
• We showcase that dropouts break the assumption of “similar cells are close to each other in space”.
• We study the impact of this on the clustering ability of commonly used scRNAseq clustering pipelines.
• Careful consideration of clustering methods and their interpretation on scRNAseq data is needed for downstream analyses.

## Graphical abstract

**Figure:**
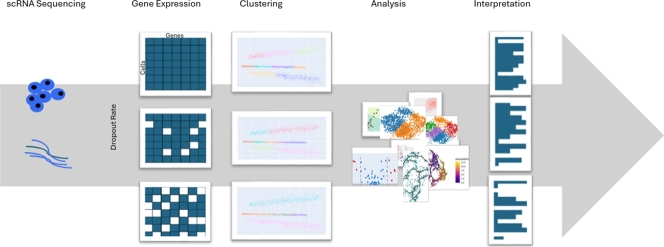

## 1 Introduction

Single Cell RNA Sequencing (scRNAseq) has made it possible to understand and investigate the biological variability between individual cells and their states. However, scRNAseq data has some unique challenges in comparison to bulk RNAseq transcriptomics. scRNAseq datasets are often of high dimensionality, not only in the number of features (genes) but also in the number of samples (cells) [14]. Furthermore, scRNAseq data show different noise profiles than bulk RNAseq data. So do scRNAseq data contain biological noise, which represents the natural heterogeneity between individual cells, which are visible with single cell technologies in contrast to bulk methods. Additionally the data contains technical noise. Differentiating between these different types of noises can be difficult [12]. One of the most challenging types of noise in scRNAseq data are dropouts [14]. Dropouts describe the phenomenon where a gene expression is observed in one cell but not in another cell of the same type, which can be a result of varying levels of mRNA between individual cells, inefficiency in capturing mRNA, or other technical and biological noise [24]. Consequently, scRNAseq transcriptomics data may have high sparsity. The sparsity varies between datasets and technologies, and zero rates of as high as 90%, have been observed [40], [38], [25]. Genes (features) with low expression counts have a higher dropout probability, suggesting that the dropout profile is not random [9], [26]. Furthermore, it is challenging to discern between “true” biological zeros and zero values caused by noise, and the term dropout is often used to describe both [14].

Many scRNAseq processing and analysis methods are based on the assumption that similar cells are close together in space with respect to the metric used. DrImpute [4] is an imputation method which uses expression values of nearby cells (clusters) to impute dropouts. mnnCorrect [7] makes use of mutual nearest neighbors for batch correction. Clustering is used to identify cell groups, which can for example be used for cell annotation or classification [36], [21], imputation [4], or differential gene expression analysis [17], [11] for example as provided by the Pegasus API [15]. Detected cell clusters are often used as an indication of cell types or subgroups of the same cell type, for example suggesting different cell states [17], [21], [5], [34].

Clustering algorithms are often evaluated based on their ability to identify homogeneous clusters based on known cell type labels, i.e., are cells in a cluster of the same type [35], [13]. These global cell type labels, however do not correctly label the heterogeneous cell population landscape available in a cell type, and therefore do not evaluate if sub-populations (e.g. as caused by cell state) are identified correctly. In addition, cell type labels may be inferred/classified based on cell clusters [36], [21], suggesting that on one hand cell labels may be used to validate a clustering algorithm, which have been created by clustering themselves and on the other hand may not identify the heterogeneous cell landscape within a population correctly, if the clustering is not identifying cell-cell relationships correctly. However, these evaluation methods based on cell types focus on global cluster homogeneity, and do not investigate if local neighborhoods/similarities are preserved (cluster stability), even though the nearest neighbor assumption is used in many downstream applications (s. above). Cluster stability assesses if cell pairs consistently appear in the same cluster, i.e., identifying whether cells in a cluster or dense region are biological neighbors. Cluster stability investigation is further complicated by a lack of true labels for biological relationship between individual cells (due to the contained noise) and hence the need for simulated data arises. During this study we will use the term global neighborhood, to describe the cell type structure of the data (i.e. are cells of the same label clustering together). In contrast we will use the term local neighborhood to describe the individual cell-cell relationships. Due to the way clustering is used in scRNAseq analyses, we define a cluster to be a local structure, with the goal to correctly identify sub-populations of the same cell type in order to capture the heterogeneous landscape of single cell studies. In this study we will use the term cluster quality to describe the global structure of the clusters, i.e. this measure will describe if within a cluster the global structure defined as cell type is conserved. The metrics used are described in section 2.6.1. Cluster stability on the other hand we will use to measure how well the local structure is preserved, which we consider to be sub cell types or cell states, i.e. the natural heterogeneous landscape of the data. The metrics used to measure cluster stability are described in section 2.6.2.

Standard scRNAseq analysis pipelines, such as the ones provided by Seurat [8] and Scanpy [30] are commonly used, and the methods provided by them are therefore widespread. The clustering pipelines in Seurat and Scanpy consist of two steps: (1) dimensionality reduction by PCA and (2) graph based clustering (via Leiden or Louvain based community detection) on a nearest neighbor graph. Both Leiden and Louvain are community detection algorithms [19]. Communities in a graph can be described as densely connected areas [19]. Both Seurat and Scanpy build the underlying network from a nearest neighbor graph and as a result the detected clusters (communities) describe dense neighborhoods on the scRNAseq data. It is assumed that individual cells in the same cluster are close in space based on the distance metric used. Therefore it is important to investigate if this assumption also holds for scRNAseq data with increasing dropout rates, since many downstream applications make the assumption that cells in a cluster are close in space/similar [4], [36], [21], [7].

Leiden clustering is based on Louvain clustering [28], making the two methods similar. The Louvain algorithm is the default algorithm used in the Seurat 5 *FindClusters()* method [8], while both the Leiden (*scanpy.tl.leiden()*) and Louvain (*scanpy.tl.louvain()*) algorithms are provided in Scanpy [30]. Thus, Leiden and Louvain based cell clustering (with default parameter settings) are commonly used clustering methodologies [39], [31], [23]. Even though this pipeline is widely used, its behavior on high dropout data, with respect to its ability to preserve local neighborhoods, has not been described in details. It is hypothesized that dropouts can lead to nearest neighbors of a cell not being its biologically true neighbors [29], but rather be caused by noise. As a result, clustering methods that are based on a nearest neighbor assumption may be impacted by the sparsity of the data.

Many downstream applications and interpretations in scRNAseq analyses built on the clustering step. Such interpretations are, for example the identification of cell sub-populations or different cell states and potential downstream applications are imputation or differential gene expression analysis [4], [17], [11]. Since clustering is a step in the scRNAseq analysis pipeline, which may have significant implications on a wide range of biological insights, we believe it is necessary to characterize the behavior of the standard pipeline with increasing noise and sparsity (dropouts), especially focusing on the ability to correctly identify cell neighborhoods.

We purposely focus the study on the most commonly used graph based clustering methods, as provided by standardized analysis packages, due to their widespread usage. We believe it is of importance to characterize behavior of used methodology first, rather than comparing a wide range of methods, that are not used due to their lack of easy access, scalability and availability to the target user group.

## 2 Methods

### 2.1 Simulated data

Since the evaluation of cluster stability requires ground truth cell-cell relationships to evaluate against, we use simulated data, and corrupt it with different rates of noise and dropouts, rather than experimental data. In order to assess how increasing levels of corruption affect the cluster stability, it is important that the baseline data to which we add corruption is the same. Therefore we first simulate expression (count) data to which we add corruption at a later stage, which is similar to the simulation methods described in [16], [37]. In order to have full control over the simulation and its parameters, we implement and adjust existing methods, as described below.

To estimate suitable sample sizes for the simulated data, that correspond to real datasets, we collected all human datasets on the Single Cell Expression Atlas [18], [3] and select our simulated data size distribution to cover 70% of the dataset size distribution. Dataset size distribution and more details are described in the supplementary methods section 1.1 and supplementary figure 1.

Baseline gene expression data is simulated with SymSim [37], which, in contrast to most simulation methods, simulate the data in a two-step process where the biological noise is simulated in the first step and technical noise is simulated in the second step. This allows insights to the true cell-cell relationship. SymSim has been rated in a recent benchmarking study of simulation methods, where it falls in tier 1 in data property estimation and biological signals and in tier 2 in scalability out of three [1]. The SymSim function and parameters used to simulate the baseline data are listed in the supplementary materials section 1.2.

#### 2.1.1 Low sample, high gene simulated data

The data sets are generated with varying numbers of cells (samples) c=[500,1000]. In addition to 20,000 genes (features), which represent the human protein coding genes [22].

#### 2.1.2 High sample, low gene simulated data

These data sets contain a large number of samples c=[5000,30000,50000]. In order to minimize computational cost, do these datasets contain a limited number of genes. The limitation of genes is in accordance with classical scRNAseq pre-processing pipelines, such as available in Scanpy [30] and Seurat [8], were (for large datasets) often only the top x most variable genes are considered. We set the number of genes to 2,000.

The datasets are labeled based on the number of samples they contain (S*number of samples*). For each sample size (number of cells) a dataset with well distinct cell types (classes) (ct=3) (S*number of samples* datasets) and a nested cell type pattern with multiple cell types and cell states (ct=8), indicated by *_O* after the S*number of samples* identifier are created. The cell type relationship trees provided to SymSim [37] are displayed in supplementary figure 1.

We define the innate noise contained in the simulated baseline data (non corrupted) as biological noise (cell heterogeneity), describing individual cell relationships and consider these as the “true cell-cell relationship pattern” during this study, which structural change with increasing dropout rate we want to investigate. We define this structure as dense neighborhoods or clusters in the data, which is in accordance with multiple scRNAseq analysis studies and pipelines. This study mainly focuses on the stability of individual clusters (per cell type) rather than cluster homogeneity and therefore the focus is on cells rather than cell types. The data sets described above will be used as baselines.

### 2.2 Real data

In addition to the simulated datasets described above, we also add two real datasets. We use the real data from [27], downloaded from their Github page (https://github.com/LuyiTian/sc_mixology). We use the 10x (sce_sc_10x_qc) and Dropseq (sce_sc_Dropseq_qc) data collection. Both datasets contain three cell lines. Due to being real data, both datasets are missing the ground truth cell-cell relationships and already contain a high number of dropouts. The 10x data contains 45% of 0 values, while the Dropseq data contains 62% of 0 values. For the real data we use the provided level of sparsity as baseline data and add further expression corruption (s. next section) to it.

### 2.3 Expression value corruption

We corrupt the data in two ways: (a) add an increasing number of zeros (dropouts) and (b) add noise.

Dropouts are added per class and low expression values have a higher probability to be set to zero (dropout), based on an adaption of the method described in [16]. A more detailed description of the method can be found in the supplementary materials section 1.2.1. Each dataset is described by the fraction of zeros it contains (its sparsity), which we vary between f=[0.1,0.2,0.3,0.4,0.5,0.6,0.7,0.8,0.9]. Additionally we apply a second dropout model from SymSim [37], by decreasing the read-depth parameter. However, due to the complexity of SymSim and the large number of variables available, it does not allow for the same control over data sparsity than the other dropout model does. Therefore we apply both dropout models (independently), where one models dropouts due to read-depth and the other focuses on expression level.

Three noise corrupted datasets are created by (1) noise is sampled from a normal distribution, (2) noise is sampled per gene based on a Gaussian distribution with mean and standard deviation of the gene, and (3) the expression value is at random replaced with another expression value of the same sample. To avoid negative expression values, the absolute value is taken after noise 1 and 2 have been added. Noise methods 1 and 2 simulate systematic noise, while noise method 3 simulates random noise, which does not take the gene expression into account. Noise is added to each expression value in the dataset with probabilities p=[0.1,0.2,0.3,0.4,0.5,0.6,0.7,0.8,0.9]. However the main investigation aspect of this study is the behavior of the default clustering pipeline with increasing sparsity (dropouts).

### 2.4 Data imputation

In order to investigate if imputation has an effect on the clustering stability the dropout corrupted data is imputed prior to clustering. Data imputation is performed with MAGIC [29] through the Scanpy API [30].

scanpy.external.pp.magic(adata, name_list="all_genes", knn=5, n_pca=None, random_state=12345, solver="approximate", copy=True)

Datasets on which imputation is performed on the dropout corrupted data are labeled with *_I* after the S*number of samples* identifier.

### 2.5 Clustering

#### 2.5.1 Random clusters

In order to investigate if the data driven detected clusters on corrupted data still contain information about the data (biological similarity), a random clustering is used as baseline for non-informative cell neighborhoods. Since the main objective of this study is to investigate the stability of cell-cell relationships or cell neighborhoods with increasing sparsity, random clusters are assigned per cell type. This simulates the scenario where the global cell type structure can be identified but where sub-cell types or cell states are of interest. This provides insight into whether data driven sub-clusters contain “biological” meaning on high dropout data, i.e. are cells in a cluster biologically similar or assigned at random. Each sample in a cell type is at random assigned to a cluster, for different numbers of clusters. The number of clusters created is based on the number of clusters identified for each dataset across all noise corruption and dropout levels by the baseline approach.

#### 2.5.2 Leiden and Louvain

Leiden/Louvain based clustering (baseline approach) is a commonly used clustering method for scRNAseq data, due to its availability in Seurat [8] and Scanpy [30]. Leiden clustering is computed with the https://github.com/MiqG/leiden_clustering API, which is based on the clustering approach implemented in Scanpy [30]. This implementation has been selected since it provides access to the underlying clustering parameters. These parameters are (n_components) the number of PCA components which controls the dimensionality reduction of the data; (n_neighbours) the number of neighbors which is used to construct the nearest neighbor graph; (metric) the metric which is used to construct the nearest neighbor graph; and (random_state) which is the seed used to approximate the nearest neighbor graph.

The baseline clustering is performed with the following parameters and non listed parameters are set to default.

pca_kws={"n_components":15} nn_kws={ "n_neighbors": 30, "metric": "euclidean", "metric_kwds": {}, "angular": False, "random_state":12345, }

To investigate the impact of the dimensionality reduction and distance parameters, the following parameter perturbations are performed for Leiden clustering: n_neighbors=[5, 30, 100], metric=["euclidean", "cosine", "manhattan"], n_components=[2, 15, 50, 100, 400]. For each parameter combinations 10 runs are performed.

Louvain clustering is computed based on the same method (https://github.com/MiqG/leiden_clustering API), where Leiden community detection is replaced by the Louvain method, and run through the NetworkX API [6].

For the uncorrupted datasets, clustering is run 100 times with different random_state parameters and its variability to the baseline (above parameters) is computed. This provides an estimate of the natural variability of the clustering methods, which is needed in order to differentiate between algorithm based variability and data driven variability.

#### 2.5.3 Other clustering methods

Since Leiden and Louvain clustering in the classical scRNAseq analysis pipelines are performed on top of PCA transformed data, K-Means and Agglomerative clustering are also applied to PCA (components = 15) transformed data. Both K-Means and Agglomerative clustering require the number of clusters to be detected as input, since this information may not be available, the number of clusters is estimated with scikit-learn's *silhouette_score* function [20], computed for cluster sizes in range 2-50. For K-Means the average number of clusters (rounded to an integer) with the best silhouette score is estimated across 10 runs.

**K-Means**

K-Means clustering is computed with the scikit-learn API [20] with default parameters and *n_init="auto"*. Algorithm variability for the uncorrupted dataset against the baseline clustering is estimated the same way as described for Leiden and Louvain based clustering, across 100 runs.

**Agglomerative**

Agglomerative clustering is computed with the scikit-learn API [20] with default parameters.

### 2.6 Cluster evaluation

Cluster quality is evaluated against the true class labels based on its homogeneity, completeness and a combined measure of the two, namely the v-measure. All three are computed with the scikit-learn API [20]. Cluster stability is assessed against clustering of the uncorrupted baseline data and measured based on the adjusted rand index (ARI), computed with the scikit-learn API [20]. Additionally, the ARI is also computed against the true cell type labels.

#### 2.6.1 Homogeneity and completeness

Let $C=\{c_{i}\}$ be the true classes from the simulation and $K=\{k_{j}\}$ be a particular clustering.

A clustering is homogeneous if all members of a cluster belong to the same class. Mathematically, homogeneity is defined as a measure *η* between 0 and 1 with 1 being a homogeneous cluster such that

$$\eta =1-\frac{H(C|K)}{H(C)},$$

where H(C|K) and H(C) denotes the entropies. Note that when H(C)=0 then the homogeneity is defined to be 1.

A clustering is complete if all members of a class belong to the same cluster. Completeness is defined as a measure *γ* between 0 and 1 with 1 being a complete cluster such that

$$\gamma =1-\frac{H(K|C)}{H(K)},$$

where H(K|C) and H(K) denotes the entropies. Note that when H(K)=0 then the completeness is defined to be 1.

The v-measure combines homogeneity and completeness through a harmonic mean such that

(1) $$V_{\beta }=\frac{(1+\beta )\eta \gamma }{\beta \eta +\gamma },$$

where we use β=1 which corresponds to an equal weighting of homogeneity and completeness and is the default in scikit-learn [20].

#### 2.6.2 Adjusted rand index

To assess cluster stability, we consider two partitionings (clusterings), where one is the clustering on the baseline data set $B=b_{i}$ and the other is a clustering on a corrupted data set $K=k_{j}$.

The rand index looks at all data point pairs (cells) and counts the number of pairs which are in agreement (either assigned to the same cluster in both clusterings *K* and *B*, or assigned to different clusters in both clusterings *K* and *B*) such that

$$\text{Rand Index}=\frac{n_{TP}+n_{TN}}{n_{\text{pairs}}},$$

where $n_{TP}$ is the number of pairs which belong to the same cluster in both clustering *K* and *B* and $n_{TN}$ is the number of pairs which belong to different clusters in both clustering *K* and *B*. Thus, the numerator is the total number of pairs in agreement.

The adjusted rand index is adjusted for chance and given by

$$\text{Adjusted Rand Index}=\frac{\text{Rand Index}-\text{Expected Rand Index}}{\text{Maximum Rand Index}-\text{Expected Rand Index}}.$$

See [10] for more information.

## 3 Results

### 3.1 Instability of clusters increases with dropout rate

**Fig. 1:**
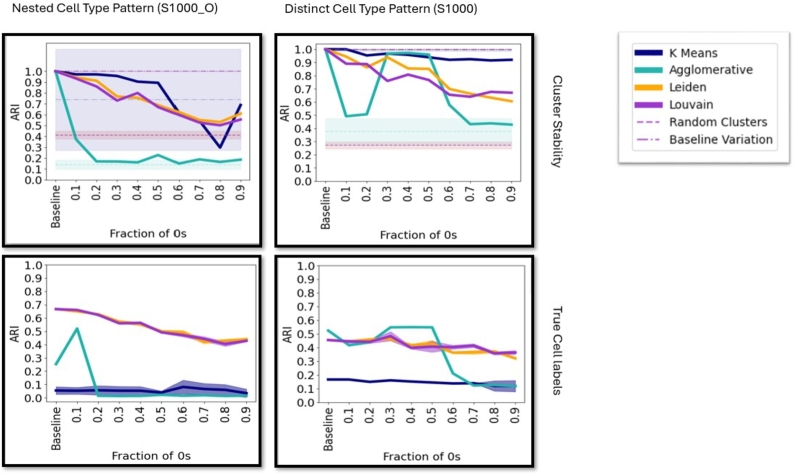
Stability between clusters with different dropout fractions on datasets with 1 000 cells (Right column: Separate cell types and Left column: nested cell type pattern, cf. supplementary table 1). Top row: Cluster stability is measured by the Adjusted Rand Index (ARI). The scores are compared to randomly assigned clusters (−−) and multiple runs of the clustering pipelines on the baseline (−.−). The shaded areas represent the standard deviation and the dotted lines the mean. All stability scores are measured against the baseline clustering, as described in the methods. Bottom row: ARI measured against the true cell labels for 100 runs of the clustering algorithm (if applicable), displaying mean and standard deviation.

Fig. 1 and supplementary figure 3 illustrate the cluster stability with increasing dropout rates in comparison to the baseline (ground truth uncorrupted data). In supplementary figure 4 the ARI, measured against the true cell type labels is displayed and in supplementary figure 5 showcases cluster stability with increasing sparsity due to the Symsim [37] sequencing depth dropout model. The cluster stability decreases continuously with the dropout rate across datasets for both Leiden and Louvain based clustering pipelines. On some datasets, cluster stability drops as low as randomly assigned clusters, indicating that local cell neighborhoods contain minimal biological information. In contrast, cluster stability stays mostly constant for K-Means and Agglomerative clustering, but this is a result of both algorithms mostly identifying the same low number of clusters for all dropout levels (for most datasets), as can be seen in their low performance against the true cell labels in supplementary figure 4. Cluster stability on data corrupted with light systematic noise (supplementary figures 6), stays consistent across different levels of noise corruption after an initial drop in comparison to the baseline. The impact of random noise on cluster stability is showcased in supplementary figure 8, which shows higher impact than dropouts on cluster stability for all four algorithms.

These results suggest that the main force driving local neighborhood instability is dropout noise, even though dropouts may not be at random [9], [26]. Therefore dropout noise should be one of the main noises considered when evaluating algorithms on their local stability. These results further imply that many of the analyses and interpretations of scRNAseq data, that make use of local neighborhoods (clusters), may be prone to capturing technical noise rather than biological relationships. Therefore interpretations of clusters should be carefully considered in combination with dropout rate of the data.

### 3.2 Cluster homogeneity is less strongly impacted by dropouts

**Fig. 2:**
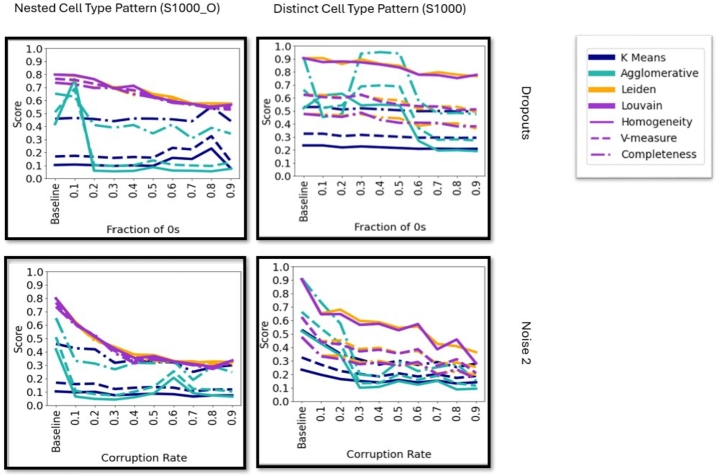
Cluster quality, measured by homogeneity (−−), v-measure (−), and completeness (..) for dropout (top row) and Noise 2 corrupted data (bottom row). The performance of the Leiden (orange), Louvain (purple), K-Means (dark blue) and Agglomerative (light blue) clustering are displayed.

Cluster homogeneity stays relatively consistent across different dropout rates with the exception of a few datasets (Fig. 2 and supplementary figure 9). Supplementary figures 10-12 showcase cluster quality for Noise 1, 2 and 3 corruption. Leiden and Louvain showcase more stability and higher overall quality scores than K-Means and Agglomerative clustering (except for Noise 3 corruption), which is likely due to the low number of clusters (supplementary figure 13) these algorithms (based on the Silhouette score) detect. Noise 1 corruption is very minor and therefore shows no significant impact on cluster quality, while Noise 2 corruption showcases a stronger impact on cluster quality than dropout corruption. These results indicate that many algorithms, evaluated based on if they identify homogeneous clusters on scRNAseq data may perform well, and therefore suggests the need to evaluate both quality and stability in bench-marking clustering algorithms for scRNAseq data analysis under dropouts.

### 3.3 Leiden cluster stability is affected by the number of PCs

Performing dimensionality reduction before clustering can reduce computational complexity as well as reduce noisiness of the data. The number of selected PCs is an important parameter in describing the underlying data. It has previously been shown that parameter selection during scRNAseq analysis pipelines is crucial and that different parameters can lead to highly different results [32], [2], [33]. Therefore it is important to evaluate the impact of the selected PC dimensions on the cluster quality and stability.

**Fig. 3:**
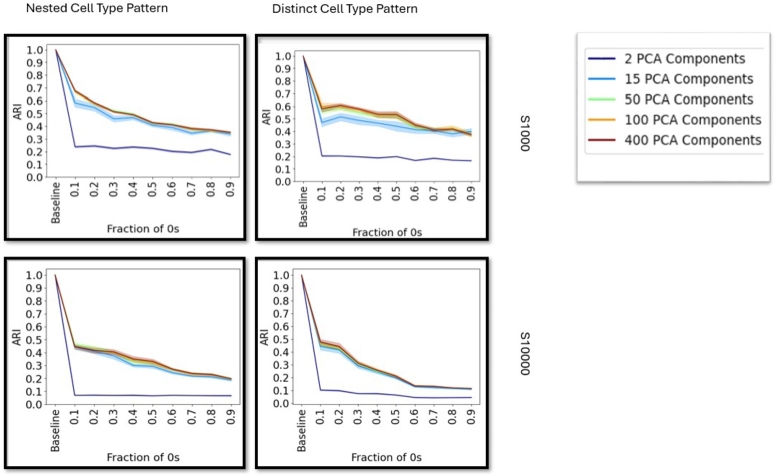
Cluster stability with different dropout fractions for the Leiden algorithm on datasets with 1 000 (top row) and 10 000 (bottom row) cells (Right column: Separate cell types and Left column: nested cell type pattern, cf. supplementary table 1) for different PCA dimensions. Cluster stability is measured by the ARI.

Cluster stability is dependent on the number of PCs selected, which is dataset dependent (Fig. 3 and supplementary figure 14). The best performing number of PCs stays mostly consistent across dropout levels. However the best performing number of dimensions is dependent on dataset size and complexity and therefore needs to be carefully evaluated beforehand. Except for dimensions=2, which performs quite low across all datasets, no strong differences can be observed, while a higher number of dimensions seems to improve cluster stability. A higher number of neighbors yields higher cluster stability, where the effect is stronger on larger datasets (supplementary figure 15). No significant difference between Euclidean and Cosine distance can be observed, however Manhattan distance shows lower cluster stability across multiple datasets (supplementary figure 16).

### 3.4 Leiden cluster quality is not affected by the Leiden clustering pipeline parameters with dropouts

**Fig. 4:**
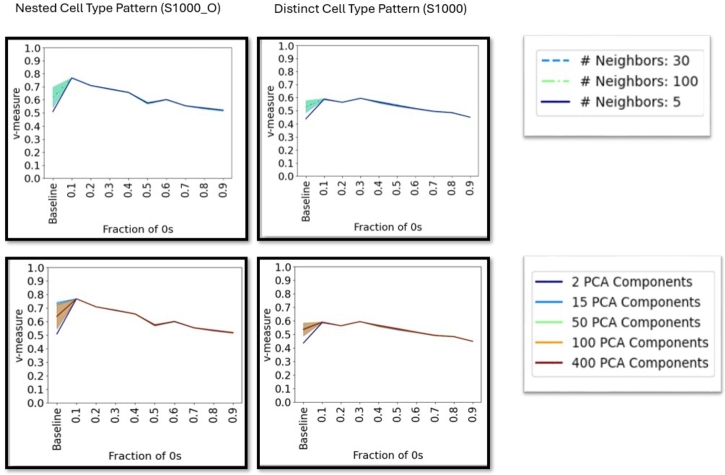
Cluster quality, measured by the v-measure with different dropout fractions for the Leiden algorithm on datasets with 1 000 cells (Right column: Separate cell types and Left column: nested cell type pattern, cf. supplementary table 1) for different numbers of nearest neighbors (top row) and PCA dimensions (bottom row). Cluster quality is measured by the v-measure.

Cluster quality is stable across different dimensionality reduction and neighborhood graph parameters for different dropout rates (Fig. 4 and supplementary figures 17-19). The only difference can be observed for the baseline data, where an effect across PCA dimensions, number of neighbors and metric can be observed (supplementary figures 17-19). However, after the initial corruption level no significant difference between the parameters can be observed.

### 3.5 Imputation does not improve cluster stability or quality

Cluster stability drops significantly after imputation with MAGIC [29] (supplementary figure 20) in comparison to the un-imputed data (supplementary figure 3). This suggests that imputation methods not only need to be evaluated in their ability to recover global cell type groups but also in their ability of recovering local neighborhoods correctly, since the incorrect imputation of data (local neighborhoods) will impact any downstream analysis (such as clustering) depending on local neighborhoods. Cluster quality stays more consistent than cluster stability, while differences between datasets can be observed.

## 4 Implication

In this study we challenged the common practice of identifying local neighborhoods in scRNAseq data by analyzing the cluster stability of commonly used clustering pipelines when dropouts are present. Our results suggest that while global cluster quality is mostly robust to dropouts (supplementary figures 25-28), local cluster stability is not. This instability has many implications for downstream application of these clusters as well as the interpretation of detected clusters. Often it is assumed that clusters represent sub-populations of cells and that cells within a cluster are similar. Our results suggest that this assumption does not hold for dropouts (supplementary figures 11-12), in contrast to systematic noise (supplementary figures 13-16). This implies that clusters on scRNAseq need to be viewed under the constrain of dropouts and as a result the interpretation of sub-populations (clusters) and their downstream application need to take this instability into account.

Currently many clustering algorithms are evaluated on their ability to detect homogeneous clusters. And while it is challenging to investigate cluster stability on real scRNAseq data, algorithms are in need to be evaluated with respect of identifying local neighborhoods under the presence of dropouts. Since clusters and cluster relationships have a large impact on downstream analyses and interpretations of scRNAseq data. This further suggests that there is a need to provide sparsity attuned clustering algorithms in a manner that they are easy to integrate with the common analysis pipelines in order to make them available to a wide range of users.

In summary our results provide insight into clustering and local neighborhoods on scRNAseq data under dropouts and have implications on how clusters on scRNAseq data need to be interpreted and suggest that further research into suitable clustering methodologies for scRNAseq data needs to be conducted, including their provisioning to the target user-group.

## CRediT authorship contribution statement

**Alisa Pavel:** Writing – review & editing, Writing – original draft, Methodology, Formal analysis, Conceptualization. **Manja Gersholm Grønberg:** Writing – review & editing, Writing – original draft, Methodology, Formal analysis. **Line H. Clemmensen:** Writing – review & editing, Supervision, Funding acquisition.

## Declaration of Competing Interest

Declarations of interest: none.

## Appendix A Supplementary material

MMC Supplementary methods and figures of the main manuscript. MMC
