# Supplementary material for: The impact of dropouts in scRNAseq dense neighborhood analysis
Source: Comput Struct Biotechnol J. 2025 Mar 24;27:1278–85. doi: 10.1016/j.csbj.2025.03.033 (PMC11992407; doi:10.1016/j.csbj.2025.03.033)
Supplement: MMC — Supplementary methods and figures of the main manuscript. [file mmc1.pdf]

The Impact of Dropouts in scRNAseq Dense Neighborhood Analysis Supplementary Materials

Alisa Pavel[1], Manja Gersholm Grønberg[1], and Line H. Clemmensen[1,2,*]

[1]Department of Applied Mathematics and Computer Science, Technical University of Denmark, 2800 Kongens Lyngby, Denmark
[2]Department of Mathematical Sciences, University of Copenhagen, Copenhagen, Denmark
[*]Corresponding Author

Contents

1 Methods **2**
1.1 Estimating scRNAseq dataset Size Distribution of Real data . . . 2
1.2 Simulated Data . . . 3
1.2.1 Dropout Model . . . 4

2 Results **6**
2.1 Instability of Clusters Increases with Dropout Rate . . . 6
2.1.1 Dropouts . . . 6
2.1.2 Cell Type Labels . . . 8
2.1.3 Sequencing Depth . . . 10
2.2 Light systematic Noise does not strongly impact cluster stability with increasing dropout rate in comparison to random noise and dropouts . . . 12
2.2.1 Noise 1 . . . 12
2.2.2 Noise 2 . . . 14
2.2.3 Noise 3 . . . 16
2.3 Cluster Homogeneity is Less Strongly Impacted by Dropouts in comparison to high systematic noise corruption as well as in graph based clustering pipelines . . . 18
2.3.1 Dropouts . . . 18
2.3.2 Noise 1 . . . 20
2.3.3 Noise 2 . . . 22
2.3.4 Noise 3 . . . 24
2.4 Number of Clusters for dropout corrupted data . . . 26

2.5 Leiden Cluster Stability is Affected by the Number of PCs 28
2.5.1 # of Components 28
2.5.2 # of Neighbors 30
2.5.3 Metric 32
2.6 Leiden Cluster Quality is not Affected by the Leiden Clustering Pipeline parameters with Dropouts 34
2.6.1 # of Components 34
2.6.2 # of Neighbors 36
2.6.3 Metric 38
2.7 Imputation does not Improve Leiden Cluster Stability or Quality 40

1 Methods

1.1 Estimating scRNAseq dataset Size Distribution of Real data

In order to select dataset sizes for our study to be reflective of dataset sizes of real data, we collected all human scRNAseq datasets from the Single Cell Expression Atlas [1, 2] (date: 01/2025). At the date of collection 159 datasets were available. We assigned each dataset to a size category of [0, 1 000), [1 000, 10 000), [10 000, 25 000), [25 000, 50 000), [50 000, 100 000), [100 000, inf) .

The size distributions are displayed in figure 1. The largest category of datasets available in the Single Cell Expression Atlas [1, 2] are datasets with less than 1000 samples, followed by datasets with less than 10 000 samples. The median sample size is 16 694. Based on this, we simulated data to cover more than 70% of the Single Cell Expression Atlas [1, 2] data set size space, by simulating data in the range 500 to 50 000 samples (s. table 1).

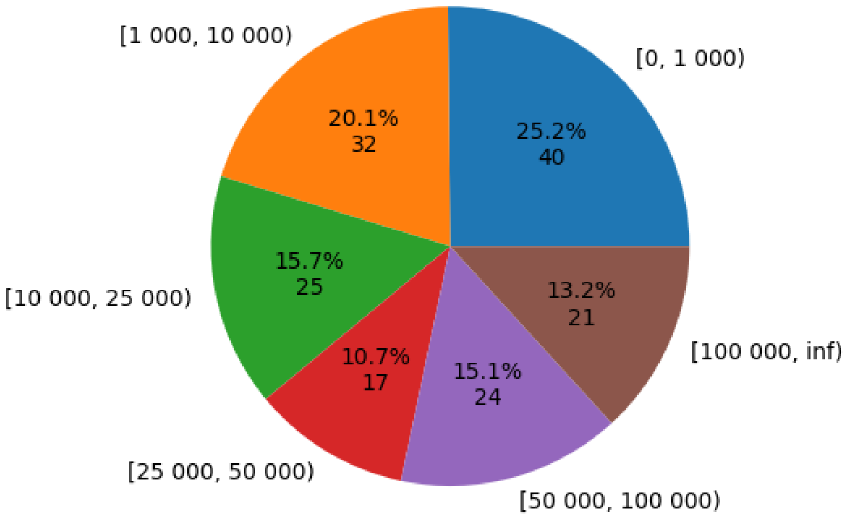

Figure 1: Dataset sample (cell) size distribution of the Single Cell Expression Atlas [1, 2]

1.2 Simulated Data

As described in the main paper, the simulated data are produced by the SymSim [3] package in R. The function and parameters used to produce the true counts with biological noise is

```
true_counts_res = SimulateTrueCounts(ncells_total=ncells,
                                     min_popsize=floor(ncells/ngroups),
                                     i_minpop=2,
                                     ngenes=ngenes,
                                     nevf=40,
                                     evf_type="discrete",
                                     n_de_evf=20,
                                     vary="s",
                                     Sigma=0.5,
                                     phyla=phyla,
                                     randseed=seed,
                                     gene_effects_sd=1,
                                     gene_effect_prob = 0.2)
```

The function and parameters used to add the technical noise to the previously simulated true counts is listed below,where the mean depth (depth_mean)

of the sequencing is varied between [10, 10^2, 10^3, 10^4, 10^5, 10^6] in order to produce different dropout levels.

```
observed_counts_res = True2ObservedCounts(true_counts_res$counts,
                                          true_counts_res$cell_meta,
                                          protocol="UMI",
                                          gene_len=gene_len,
                                          depth_mean = dm,
                                          depth_sd = 3e3)
```

1.2.1 Dropout Model

Expression values are normalized per sample (cell) in order to keep the individual gene expression structures for each cell. A expression value is set to 0 if it's value is lower than a randomly drawn float [0,1], where expression values are previously multiplied by a user provided float (after normalization), which controls the rate at which values are set to 0.

Data Set ID	#Cells	#Classes	#Genes	Cell Type Relationship	Imputed?
S500	500	3	20 000	distinct	no
S500_O	500	8	20 000	nested	no
S500_I	500	3	20 000	distinct	yes
S500_O_I	500	8	20 000	nested	yes
S1000	1 000	3	20 000	distinct	no
S1000_O	1 000	8	20 000	nested	no
S1000_I	1 000	3	20 000	distinct	yes
S1000_O_I	1 000	8	20 000	nested	yes
S10000	10 000	3	2 000	distinct	no
S10000_O	10 000	8	2 000	nested	no
S10000_I	10 000	3	2 000	distinct	yes
S10000_O_I	10 000	8	2 000	nested	yes
S30000	30 000	3	2 000	distinct	no
S30000_O	30 000	8	2 000	nested	no
S30000_I	30 000	3	2 000	distinct	yes
S30000_O_I	30 000	8	2 000	nested	yes
S50000	50 000	3	2 000	distinct	no
S50000_O	50 000	8	2 000	nested	no
S50000_I	50 000	3	2 000	distinct	yes
S50000_O_I	50 000	8	2 000	nested	yes
SC Mixology 10x	902	3	16 468	distinct	no
SC Mixology Dropseq	225	3	15 127	distinct	no

Table 1: Data sets, identifiers and characteristics.

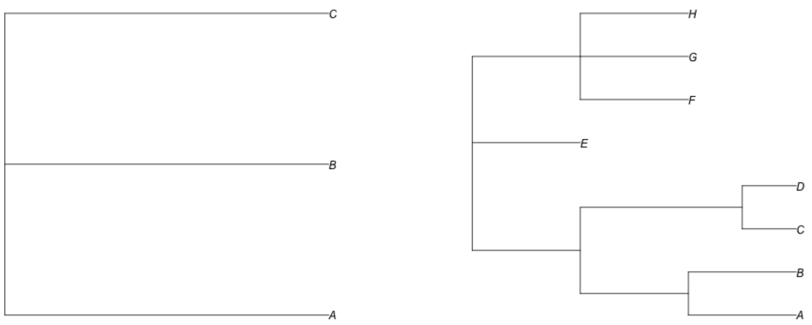

Figure 2: Relationship tree of the simulated distinct (left) and nested data (right) as provided to Symsim [3]

2 Results

2.1 Instability of Clusters Increases with Dropout Rate

2.1.1 Dropouts

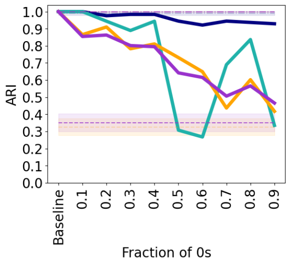

(a) S500

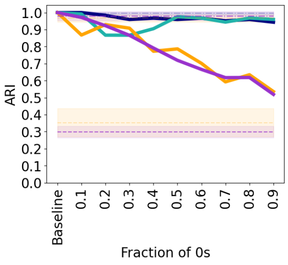

(b) S500_O

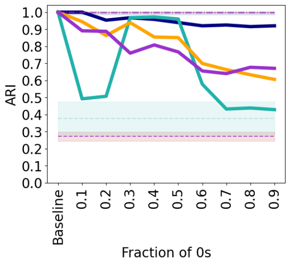

(c) S1000

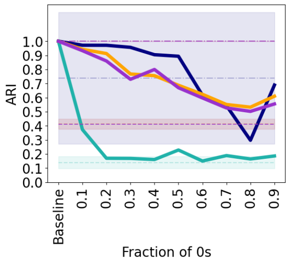

(d) S1000_O

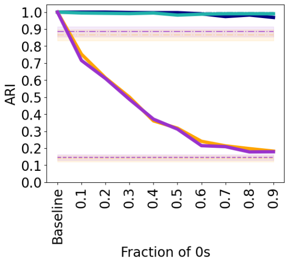

(e) S10000

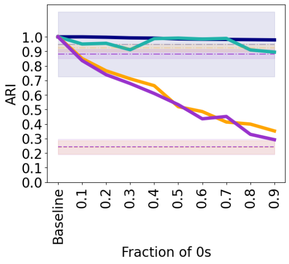

(f) S10000_O

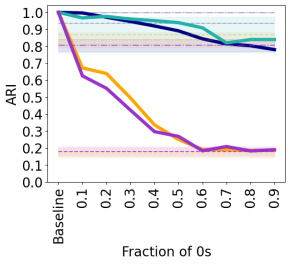

(g) S30000

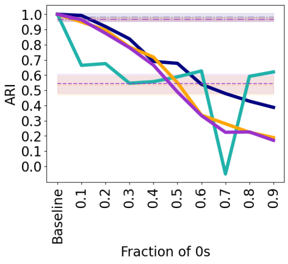

(h) S30000_O

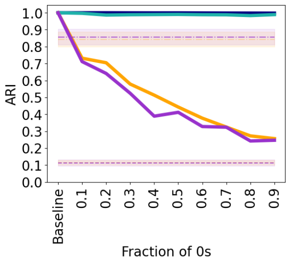

(i) S50000

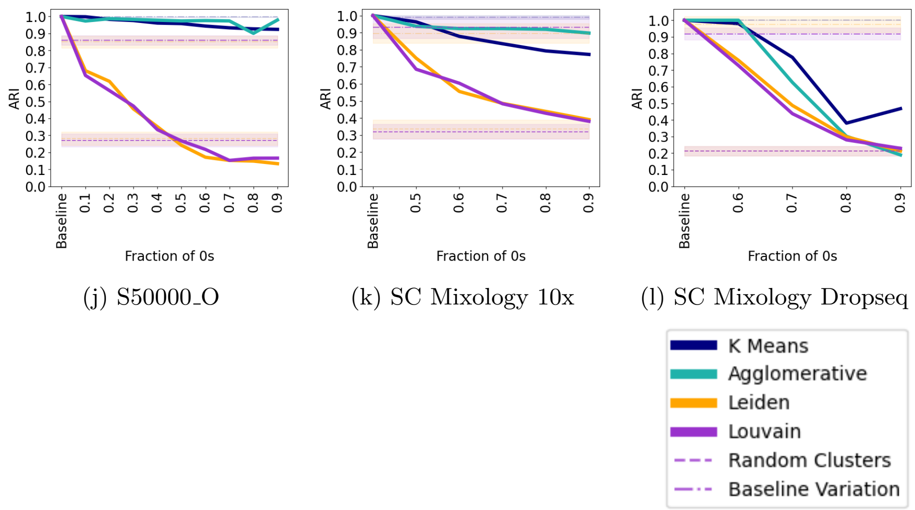

(j) S50000_O (k) SC Mixology 10x (l) SC Mixology Dropseq

Figure 3: Adjusted Rand Index (cluster stability) between clusters with different dropout fractions in comparison to random assigned clusters and variability of the Leiden (orange), Louvain (purple), K-Means (dark blue) and Agglomerative (light blue) clustering algorithm. The variability of random assigned clusters to the baseline clustering is indicated with $--$ and the variability of the clustering algorithm on the uncorrupted data against the base line clustering (where applicable) is showcased with $-.-$.

2.1.2 Cell Type Labels

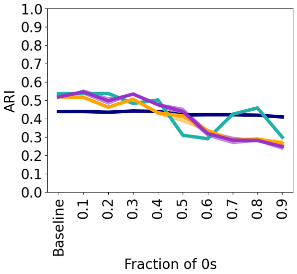

(a) S500

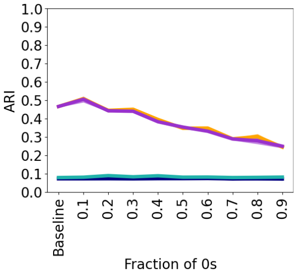

(b) S500_O

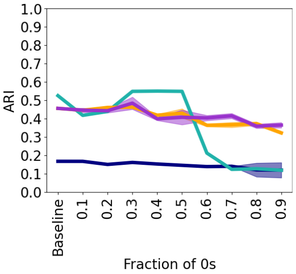

(c) S1000

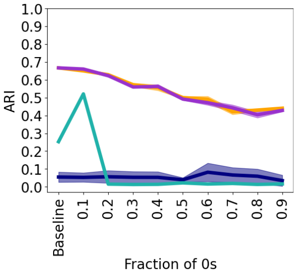

(d) S1000_O

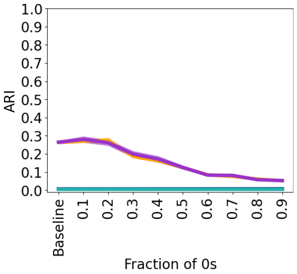

(e) S10000

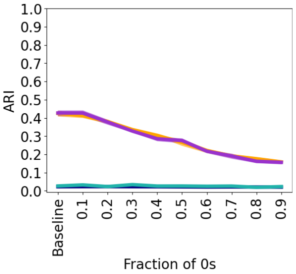

(f) S10000_O

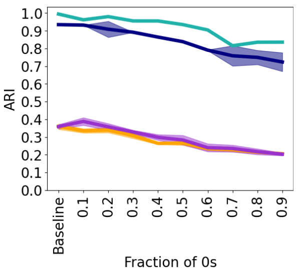

(g) S30000

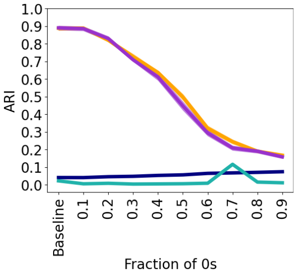

(h) S30000_O

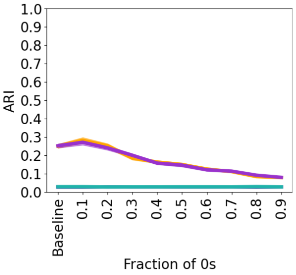

(i) S50000

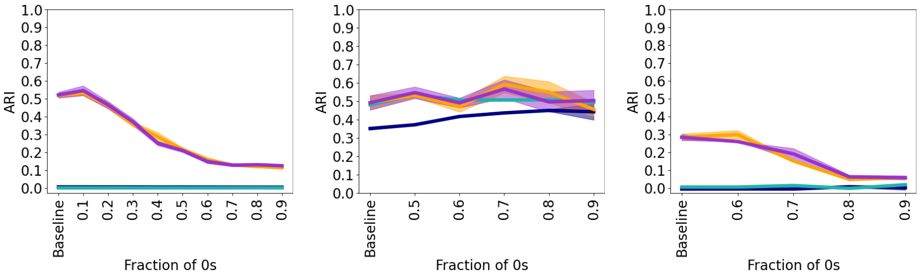

(j) S50000_O (k) SC Mixology 10x (l) SC Mixology Dropseq

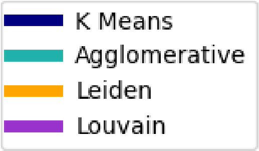

Figure 4: Adjusted Rand Index (cluster stability) between clusters with different dropout fractions in comparison to the true cell labels for the Leiden (orange), Louvain (purple), K-Means (dark blue) and Agglomerative (light blue) clustering algorithm. Where applicable the mean and std of 100 runs is indicated.

2.1.3 Sequencing Depth

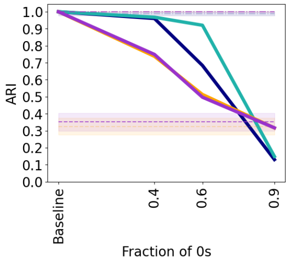

(a) S500

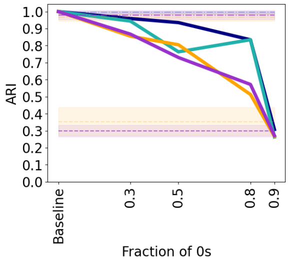

(b) S500_O

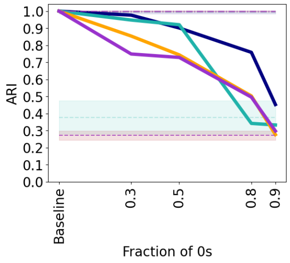

(c) S1000

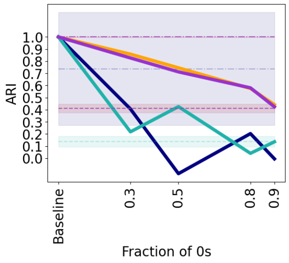

(d) S1000_O

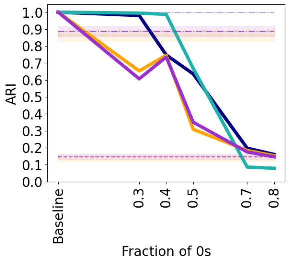

(e) S10000

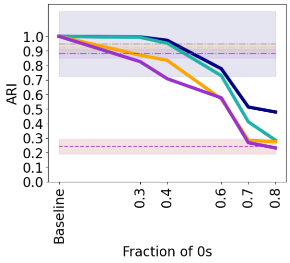

(f) S10000_O

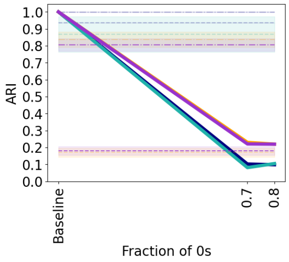

(g) S30000

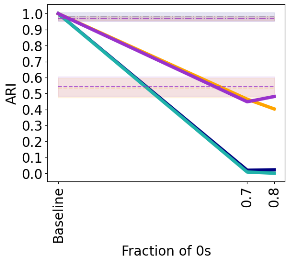

(h) S30000_O

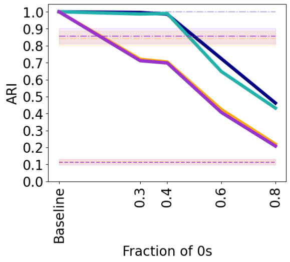

(i) S50000

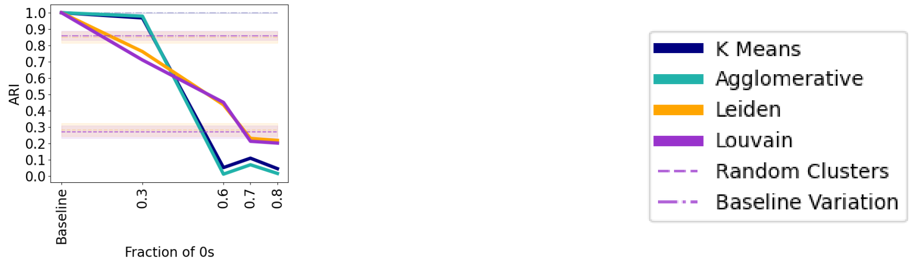

(j) S50000_O

Figure 5: Adjusted Rand Index (cluster stability) between clusters with different dropout fractions due to reduced sequencing depth, in comparison to clustering on the baseline data for the Leiden (orange), Louvain (purple), K-Means (dark blue) and Agglomerative (light blue) clustering algorithm. The variability of random assigned clusters to the baseline clustering is indicated with $--$ and the variability of the clustering algorithm on the uncorrupted data against the base line clustering (where applicable) is showcased with $-.-$.

2.2 Light systematic Noise does not strongly impact cluster stability with increasing dropout rate in comparison to random noise and dropouts

2.2.1 Noise 1

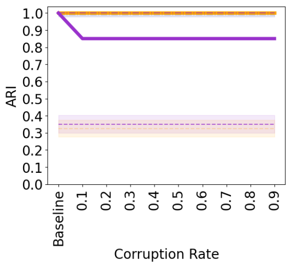

(a) S500

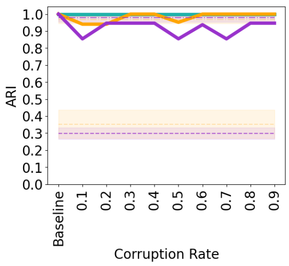

(b) S500_O

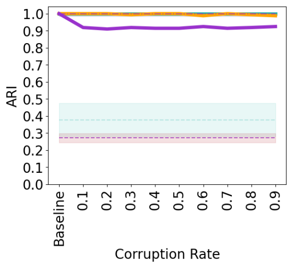

(c) S1000

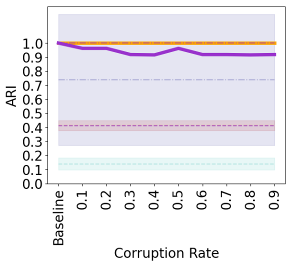

(d) S1000_O

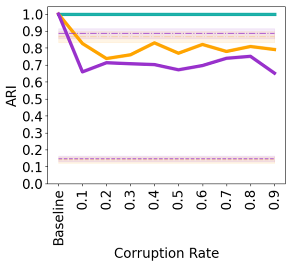

(e) S10000

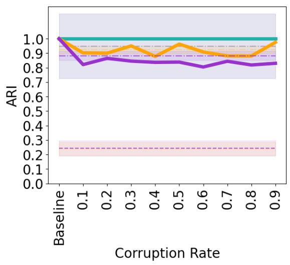

(f) S10000_O

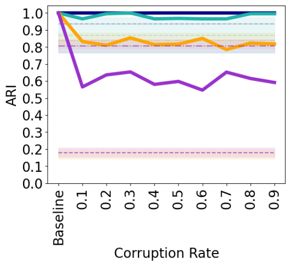

(g) S30000

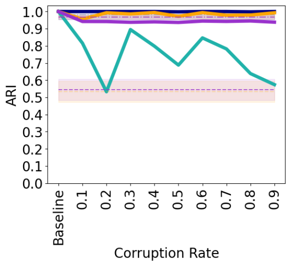

(h) S30000_O

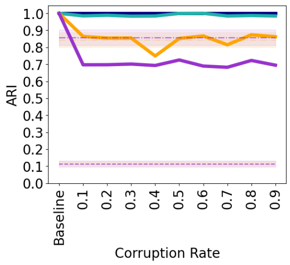

(i) S50000

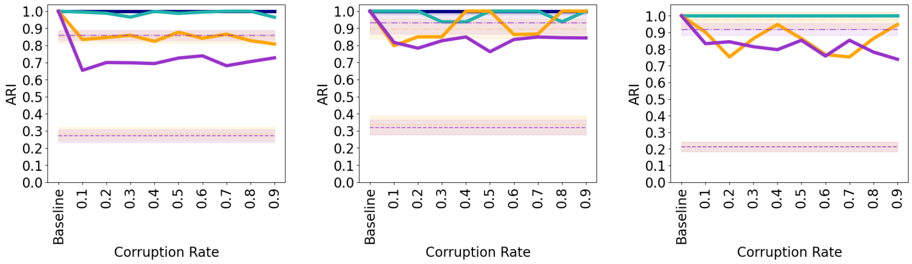

(j) S50000_O (k) SC Mixology 10x (l) SC Mixology Dropseq

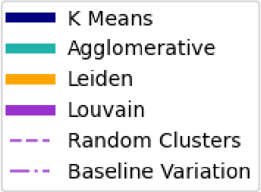

Figure 6: Adjusted Rand Index (cluster stability) between clusters with different Noise 1 corruption rates in comparison to random assigned clusters and variability of the Leiden (orange), Louvain (purple), K-Means (dark blue) and Agglomerative (light blue) clustering algorithm. The variability of random assigned clusters to the baseline clustering is indicated with $--$ and the variability of the clustering algorithm on the uncorrupted data against the base line clustering (where applicable) is showcased with $-.-$.

2.2.2 Noise 2

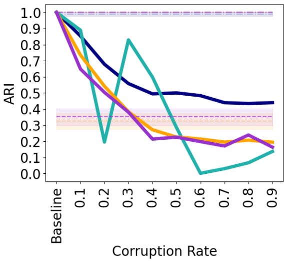

(a) S500

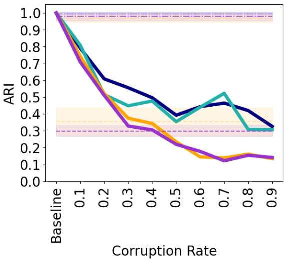

(b) S500_O

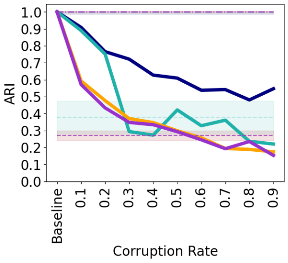

(c) S1000

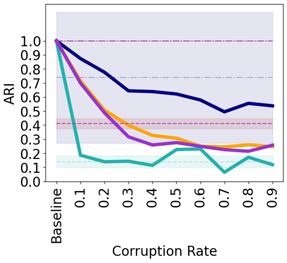

(d) S1000_O

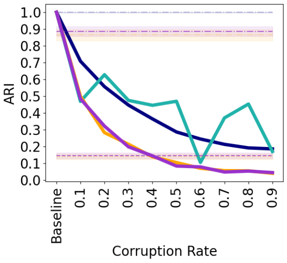

(e) S10000

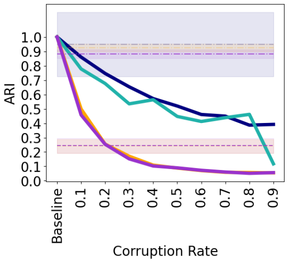

(f) S10000_O

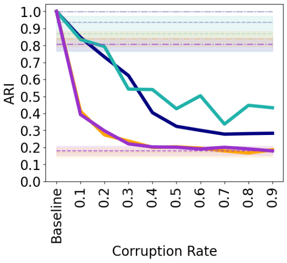

(g) S30000

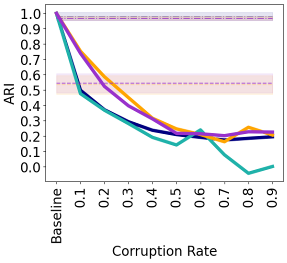

(h) S30000_O

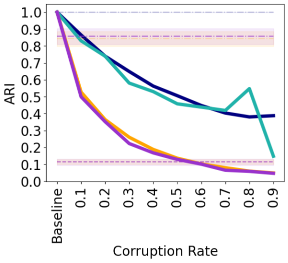

(i) S50000

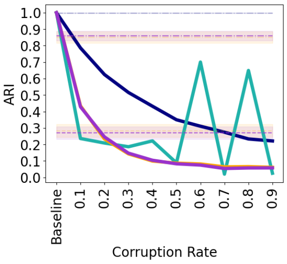

(j) S50000_O

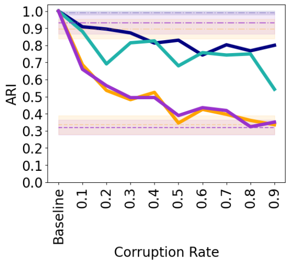

(k) SC Mixology 10x

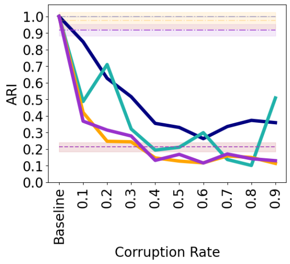

(l) SC Mixology Dropseq

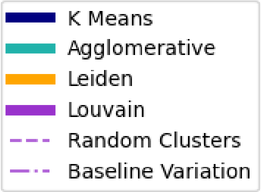

Figure 7: Adjusted Rand Index (cluster stability) between clusters with different Noise 2 corruption rates in comparison to random assigned clusters and variability of the Leiden (orange), Louvain (purple), K-Means (dark blue) and Agglomerative (light blue) clustering algorithm. The variability of random assigned clusters to the baseline clustering is indicated with −− and the variability of the clustering algorithm on the uncorrupted data against the base line clustering (where applicable) is showcased with −.−.

2.2.3 Noise 3

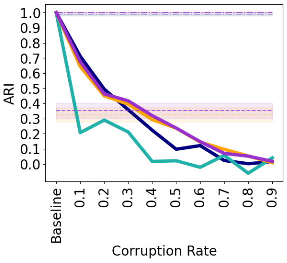

(a) S500

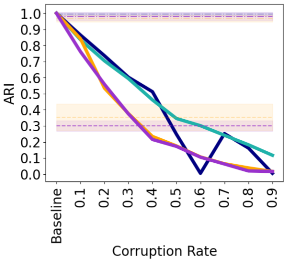

(b) S500_O

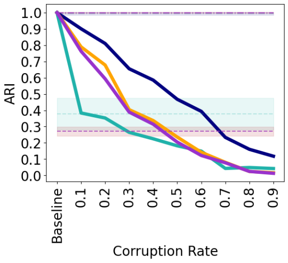

(c) S1000

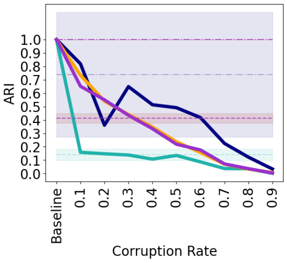

(d) S1000_O

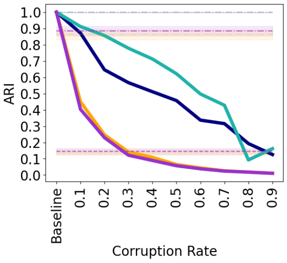

(e) S10000

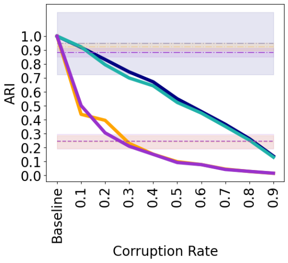

(f) S10000_O

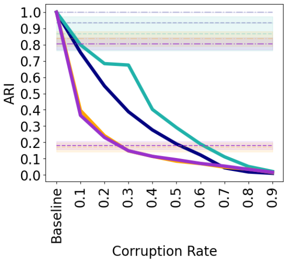

(g) S30000

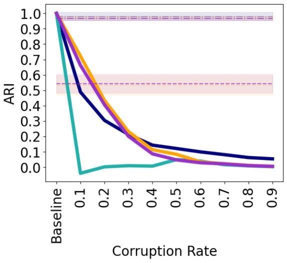

(h) S30000_O

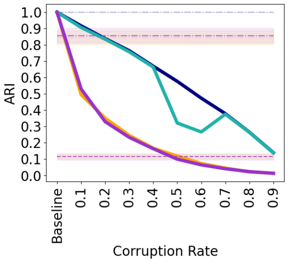

(i) S50000

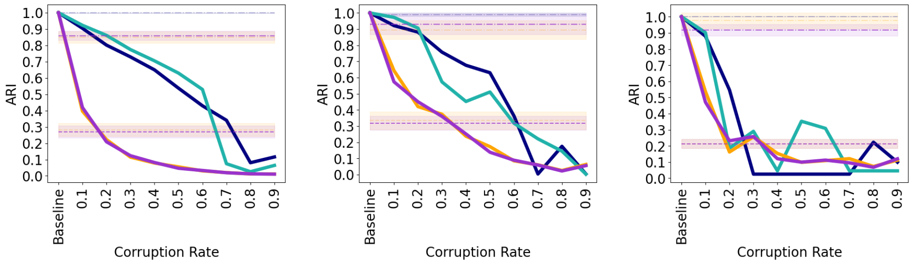

(j) S50000_O (k) SC Mixology 10x (l) SC Mixology Dropseq

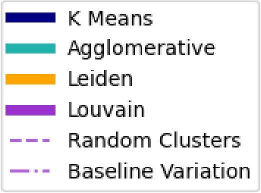

Figure 8: Adjusted Rand Index (cluster stability) between clusters with different Noise 3 corruption rates in comparison to random assigned clusters and variability of the Leiden (orange), Louvain (purple), K-Means (dark blue) and Agglomerative (light blue) clustering algorithm. The variability of random assigned clusters to the baseline clustering is indicated with $--$ and the variability of the clustering algorithm on the uncorrupted data against the base line clustering (where applicable) is showcased with $-.-$.

2.3 Cluster Homogeneity is Less Strongly Impacted by Dropouts in comparison to high systematic noise corruption as well as in graph based clustering pipelines

2.3.1 Dropouts

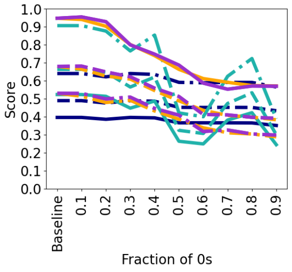

(a) S500

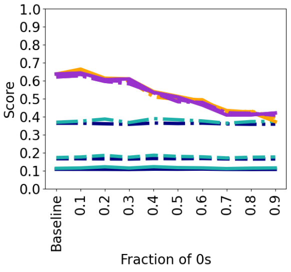

(b) S500_O

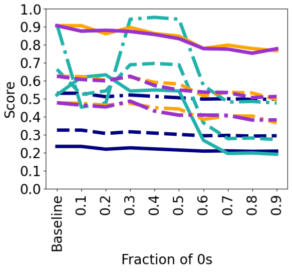

(c) S1000

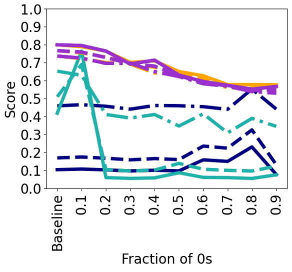

(d) S1000_O

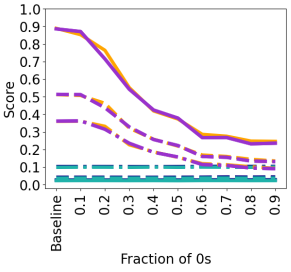

(e) S10000

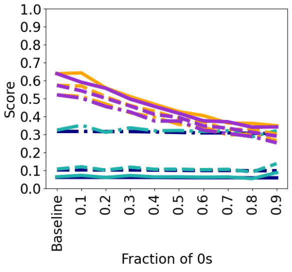

(f) S10000_O

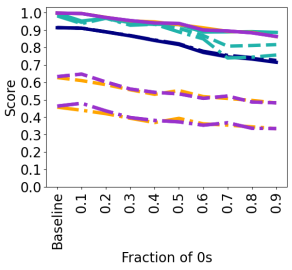

(g) S30000

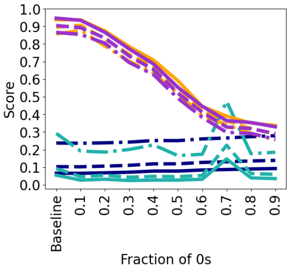

(h) S30000_O

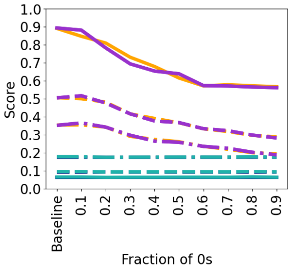

(i) S50000

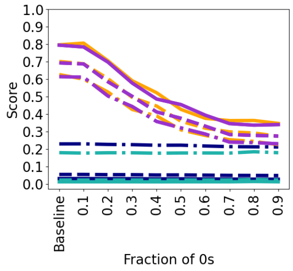

(j) S50000_O

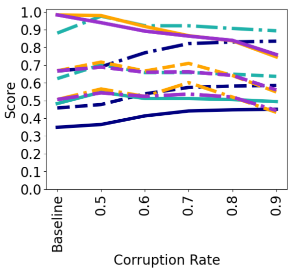

(k) SC Mixology 10x

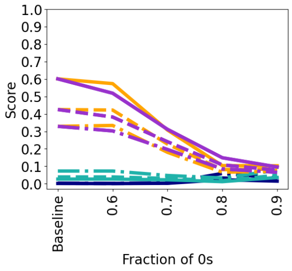

(l) SC Mixology Dropseq

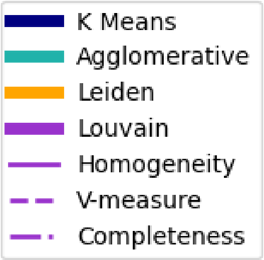

Figure 9: Cluster quality with increasing dropout fractions the Leiden (orange), Louvain (purple), K-Means (dark blue) and Agglomerative (light blue) clustering algorithms. Cluster quality is measured by homogeneity (−), completeness (−.−) and the v-measure (−−).

2.3.2 Noise 1

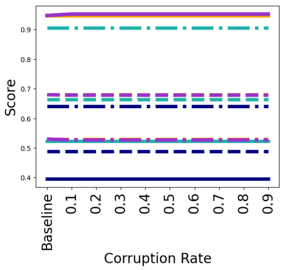

(a) S500

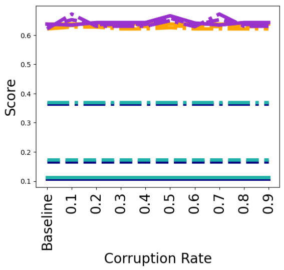

(b) S500_O

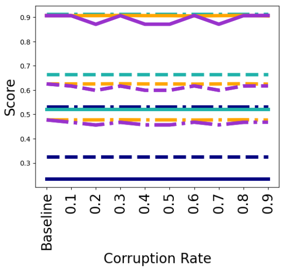

(c) S1000

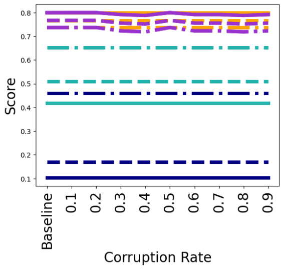

(d) S1000_O

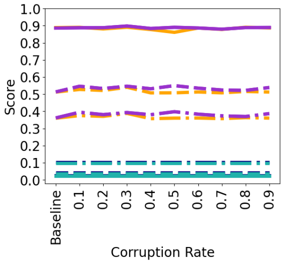

(e) S10000

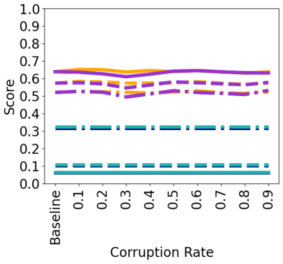

(f) S10000_O

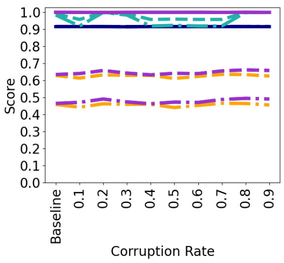

(g) S30000

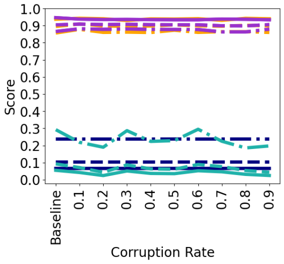

(h) S30000_O

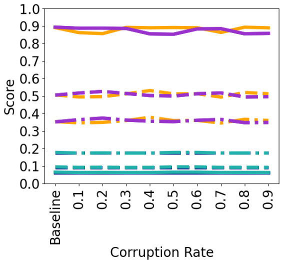

(i) S50000

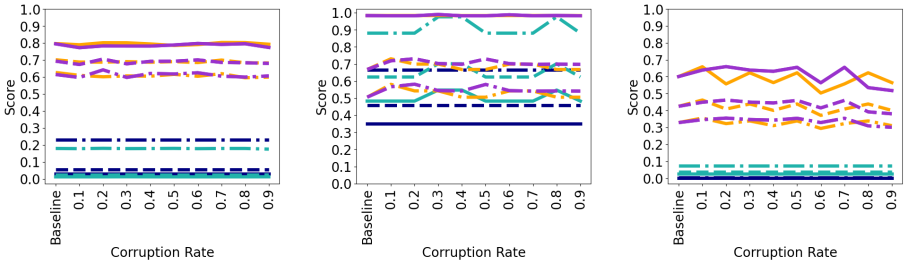

(j) S50000_O (k) SC Mixology 10x (l) SC Mixology Dropseq

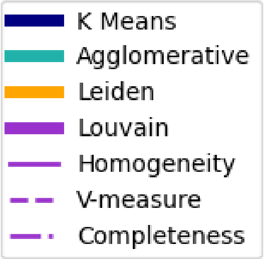

Figure 10: Cluster quality, measured by homogeneity (−), completeness (−.−) and the v-measure (−−) against the true class labels with increasing Noise 1 corruption for the Leiden (orange), Louvain (purple), K-Means (dark blue) and Agglomerative (light blue) clustering algorithms.

2.3.3 Noise 2

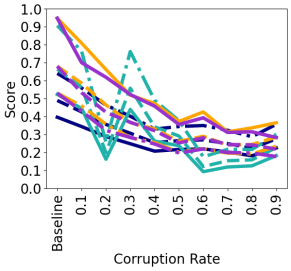

(a) S500

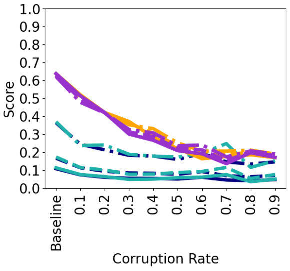

(b) S500_O

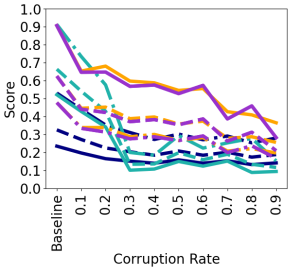

(c) S1000

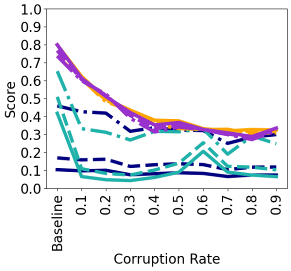

(d) S1000_O

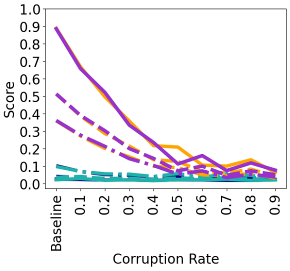

(e) S10000

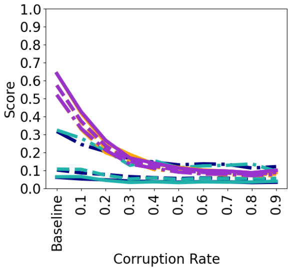

(f) S10000_O

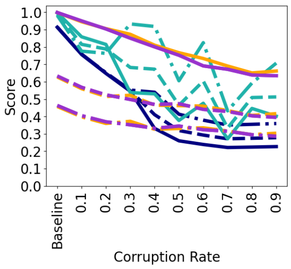

(g) S30000

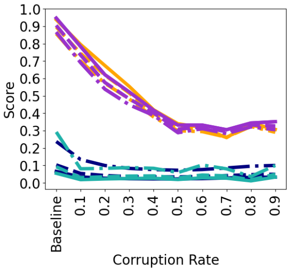

(h) S30000_O

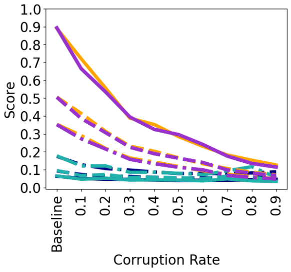

(i) S50000

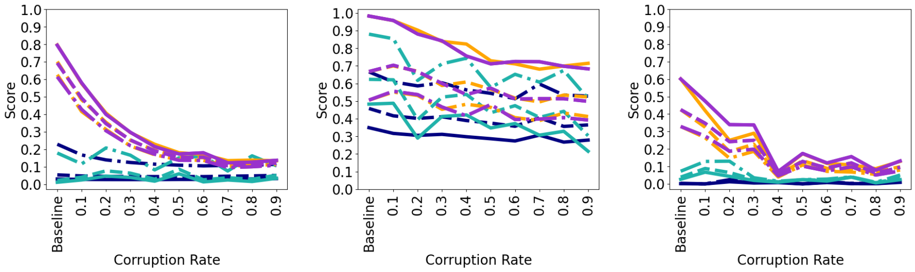

(j) S50000_O (k) SC Mixology 10x (l) SC Mixology Dropseq

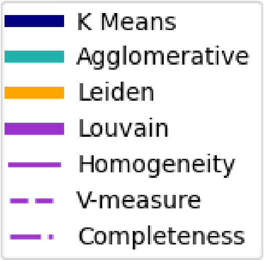

Figure 11: Cluster quality, measured by homogeneity (–), completeness (–.–) and the v-measure (– –) against the true class labels with increasing Noise 2 corruption for the Leiden (orange), Louvain (purple), K-Means (dark blue) and Agglomerative (light blue) clustering algorithms.

2.3.4 Noise 3

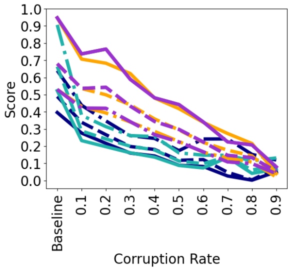

(a) S500

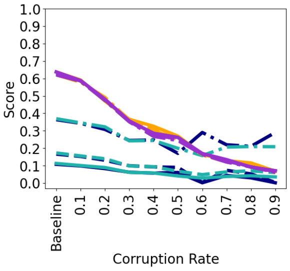

(b) S500_O

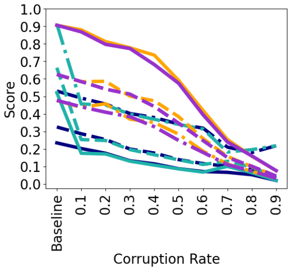

(c) S1000

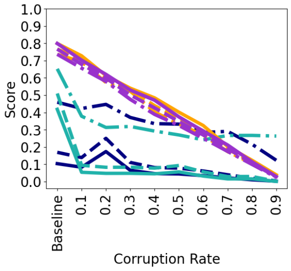

(d) S1000_O

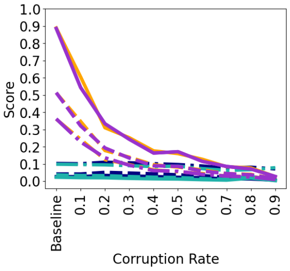

(e) S10000

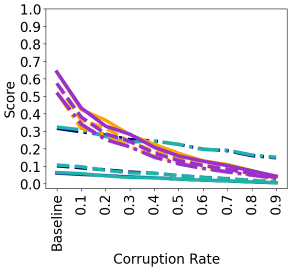

(f) S10000_O

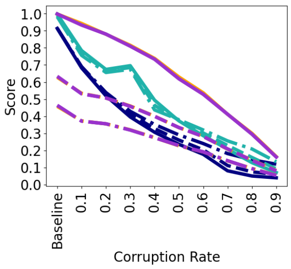

(g) S30000

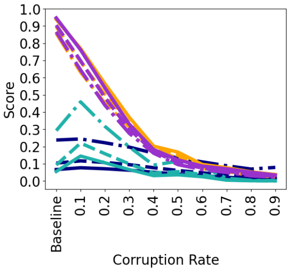

(h) S30000_O

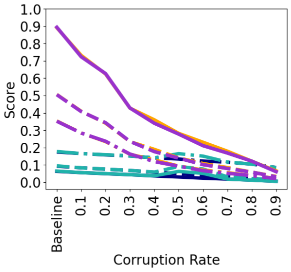

(i) S50000

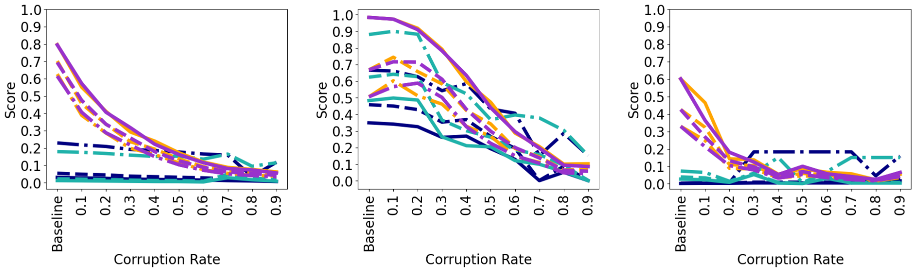

(j) S50000_O (k) SC Mixology 10x (l) SC Mixology Dropseq

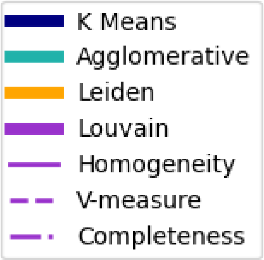

Figure 12: Cluster quality, measured by homogeneity (–), completeness (–.–) and the v-measure (– –) against the true class labels with increasing Noise 3 corruption for the Leiden (orange), Louvain (purple), K-Means (dark blue) and Agglomerative (light blue) clustering algorithms.

2.4 Number of Clusters for dropout corrupted data

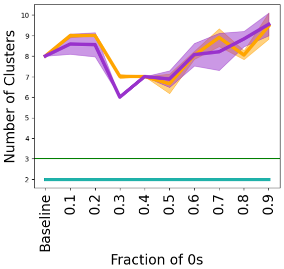

(a) S500

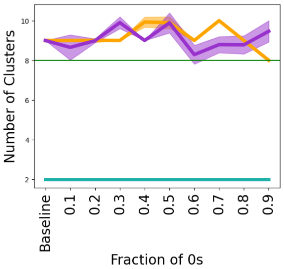

(b) S500_O

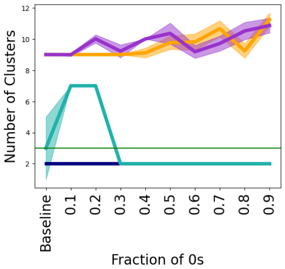

(c) S1000

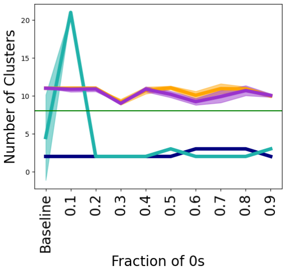

(d) S1000_O

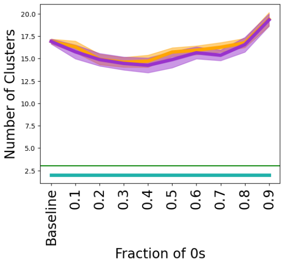

(e) S10000

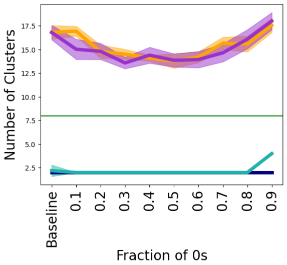

(f) S10000_O

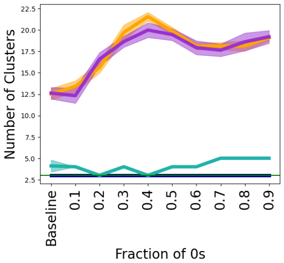

(g) S30000

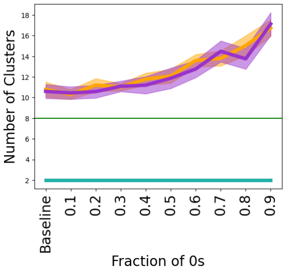

(h) S30000_O

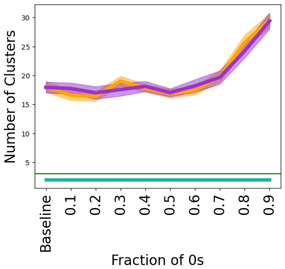

(i) S50000

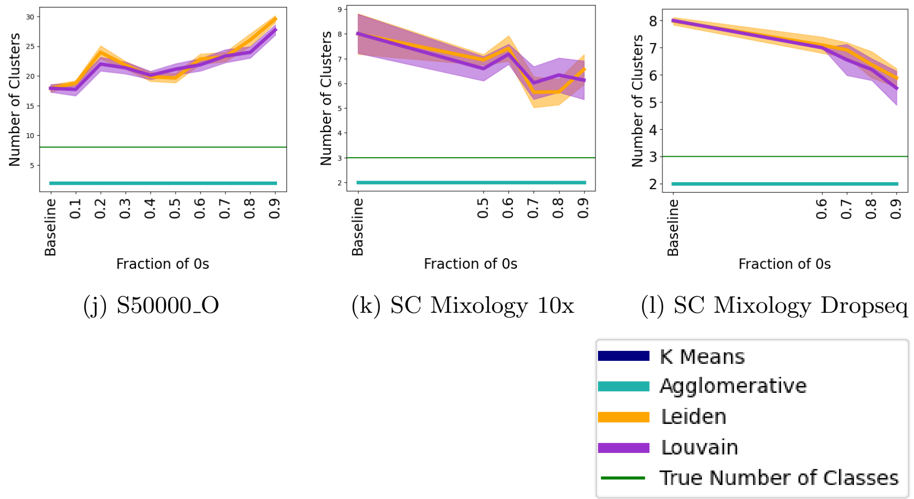

(j) S50000_O (k) SC Mixology 10x (l) SC Mixology Dropseq

Figure 13: Number of Clusters detected with varying levels dropouts for Leiden, Louvain, KMeans and Agglomerative Clustering. The shaded parts indicate the mean and standard-deviation of clusters detected across 100 runs (if applicable).

2.5 Leiden Cluster Stability is Affected by the Number of PCs

2.5.1 # of Components

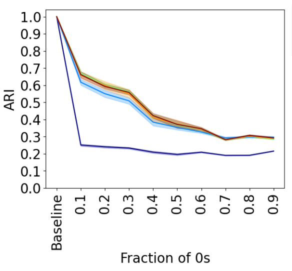

(a) S500

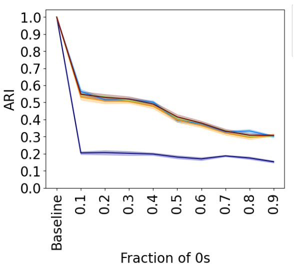

(b) S500_O

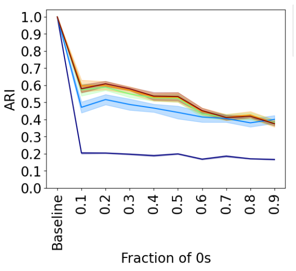

(c) S1000

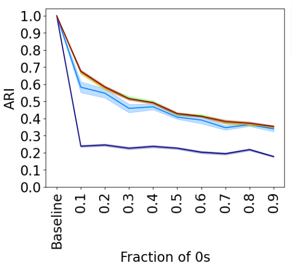

(d) S1000_O

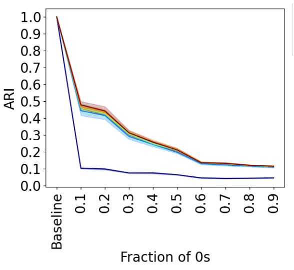

(e) S10000

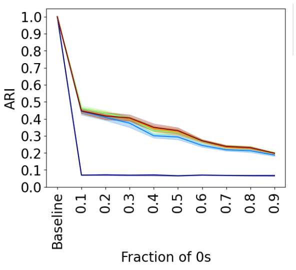

(f) S10000_O

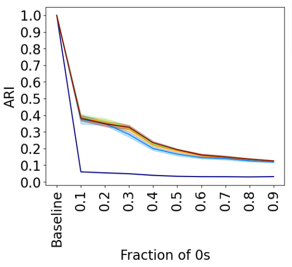

(g) S30000

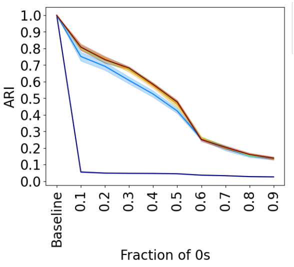

(h) S30000_O

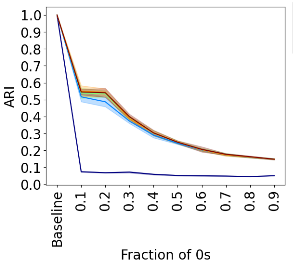

(i) S50000

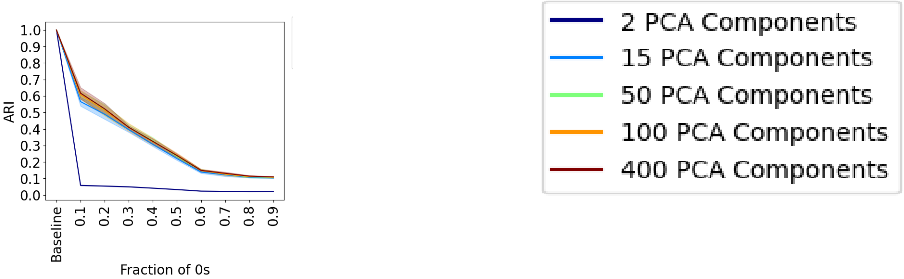

(j) S50000_O

Figure 14: ARI for an increasing number of dropouts against the baseline clustering for a different number of PCA components provided to Leiden clustering algorithm. Leiden clustering is performed with metric=euclidean and number of neighbors = 5. Mean and standard deviation of 10 runs are indicated.

2.5.2 # of Neighbors

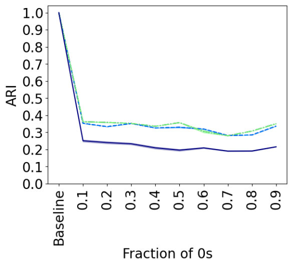

(a) S500

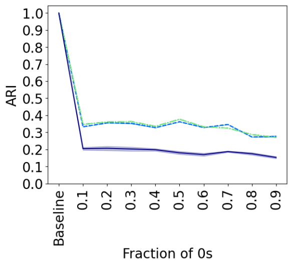

(b) S500_O

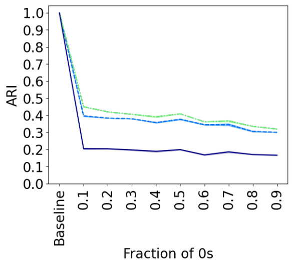

(c) S1000

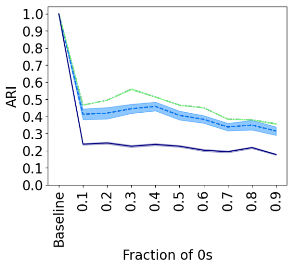

(d) S1000_O

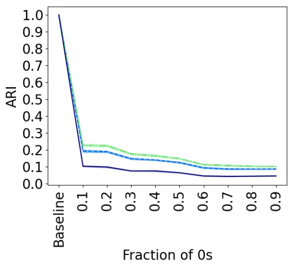

(e) S10000

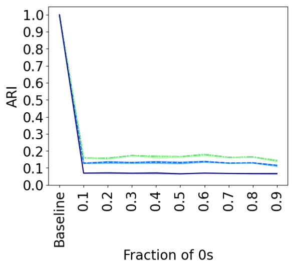

(f) S10000_O

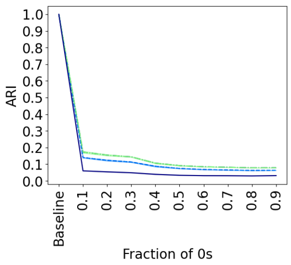

(g) S30000

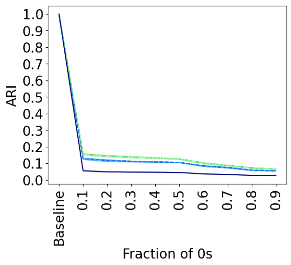

(h) S30000_O

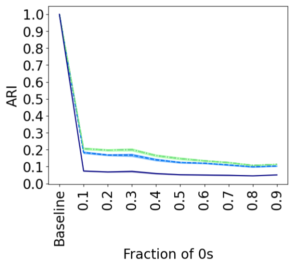

(i) S50000

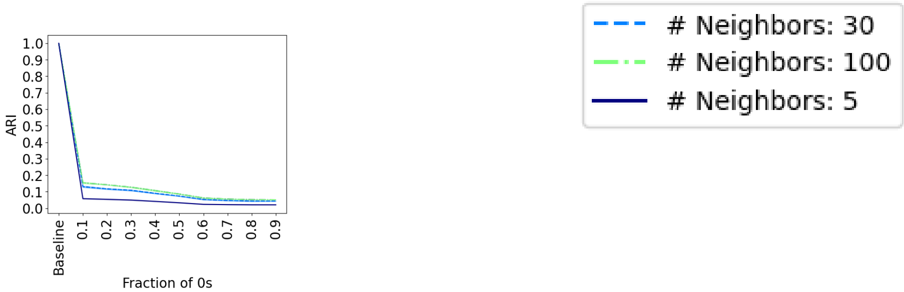

(j) S50000_O

Figure 15: ARI for an increasing number of dropouts against the baseline clustering for different Leiden n_neighbors parameters. Leiden clustering is performed with metric=euclidean and number of dimensions = 2. Mean and standard deviation of 10 runs are indicated.

2.5.3 Metric

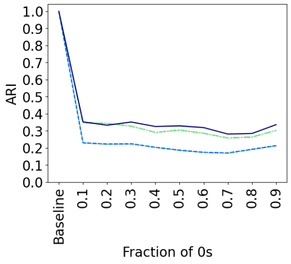

(a) S500

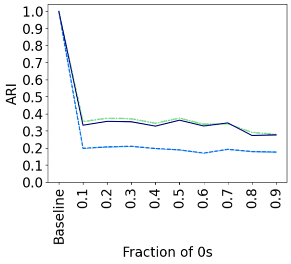

(b) S500_O

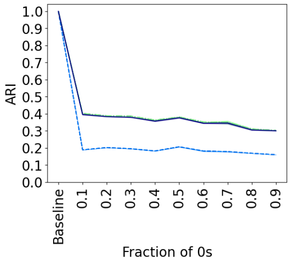

(c) S1000

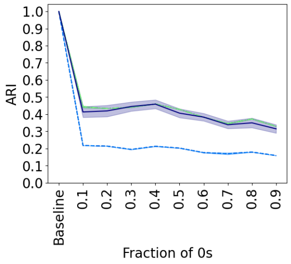

(d) S1000_O

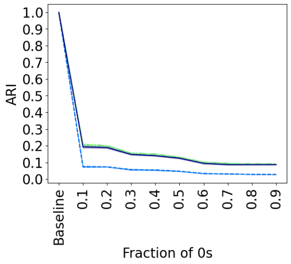

(e) S10000

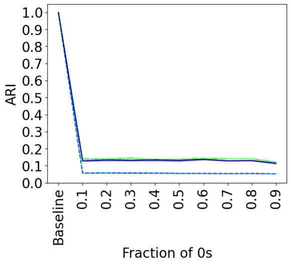

(f) S10000_O

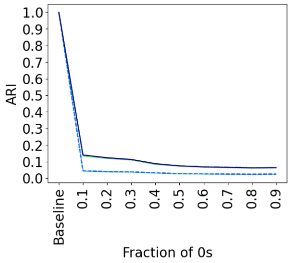

(g) S30000

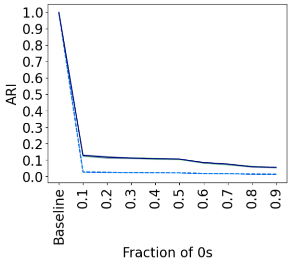

(h) S30000_O

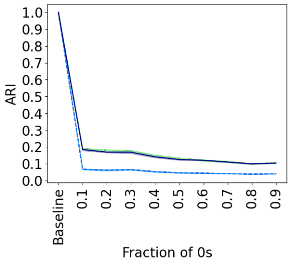

(i) S50000

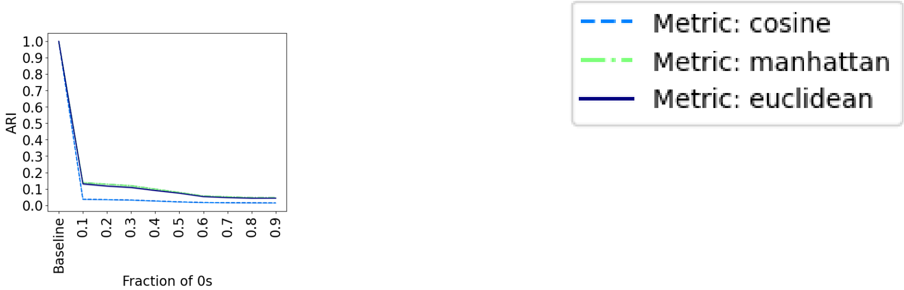

(j) S50000_O

Figure 16: ARI for an increasing number of dropouts against the baseline clustering for different Leiden metric parameters. Leiden clustering is performed with number of neighbors = 5 and number of dimensions = 2. Mean and standard deviation of 10 runs are indicated.

2.6 Leiden Cluster Quality is not Affected by the Leiden Clustering Pipeline parameters with Dropouts

2.6.1 # of Components

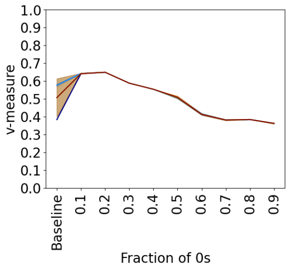

(a) S500

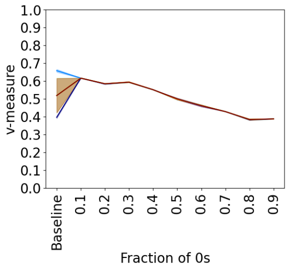

(b) S500_O

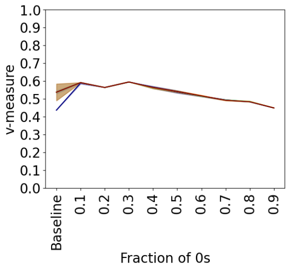

(c) S1000

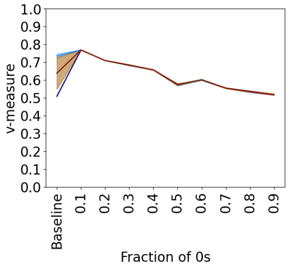

(d) S1000_O

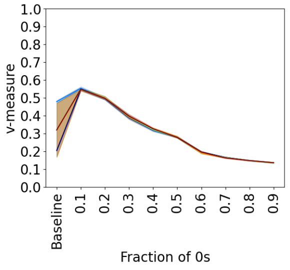

(e) S10000

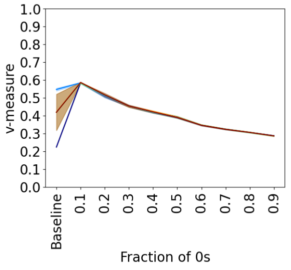

(f) S10000_O

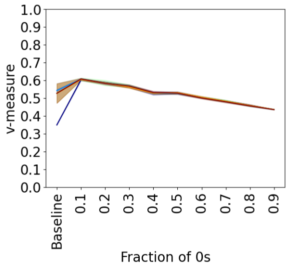

(g) S30000

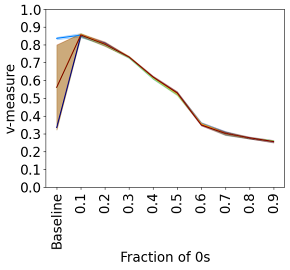

(h) S30000_O

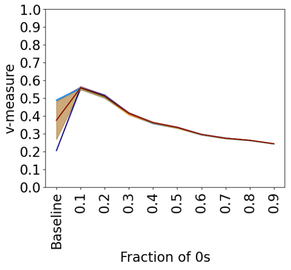

(i) S50000

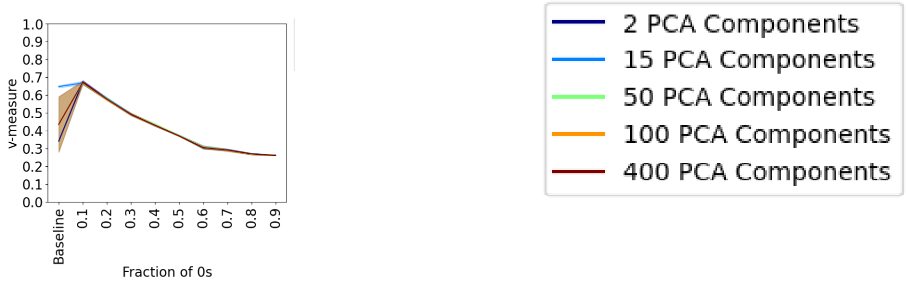

(j) S50000_O

Figure 17: V-measure for an increasing number of dropouts against the baseline clustering for a different number of PCA components provided to Leiden clustering algorithm. Leiden clustering is performed with metric=euclidean and number of neighbors = 5. Mean and standard deviation of 10 runs are indicated.

2.6.2 # of Neighbors

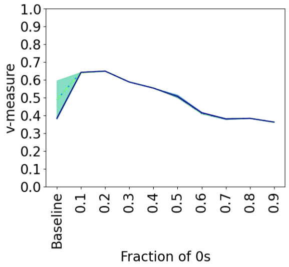

(a) S500

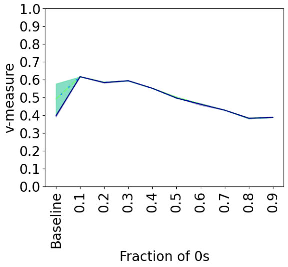

(b) S500_O

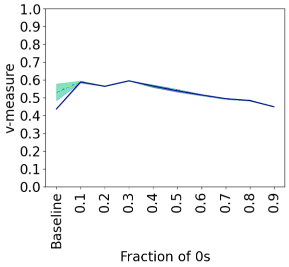

(c) S1000

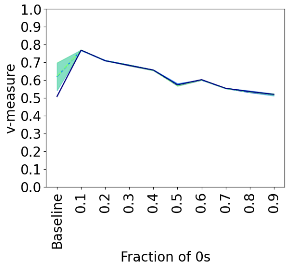

(d) S1000_O

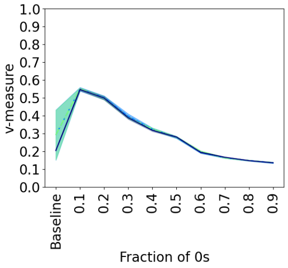

(e) S10000

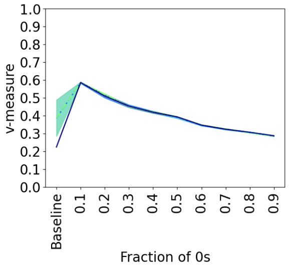

(f) S10000_O

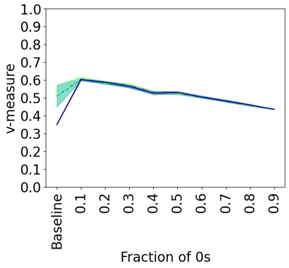

(g) S30000

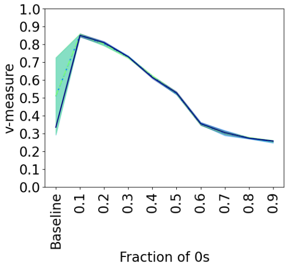

(h) S30000_O

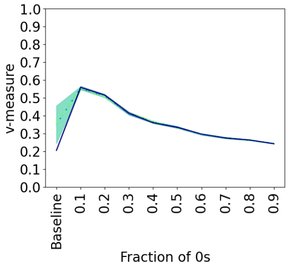

(i) S50000

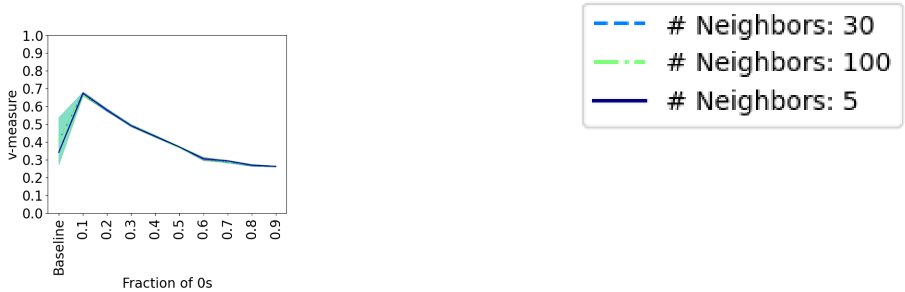

(j) S50000_O

Figure 18: V-measure for an increasing number of dropouts against the baseline clustering for different Leiden n_neighbors parameters. Leiden clustering is performed with metric=euclidean and number of dimensions = 2. Mean and standard deviation of 10 runs are indicated.

2.6.3 Metric

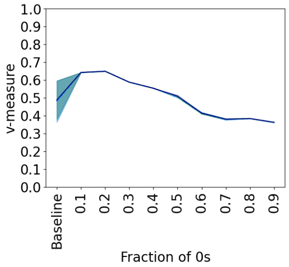

(a) S500

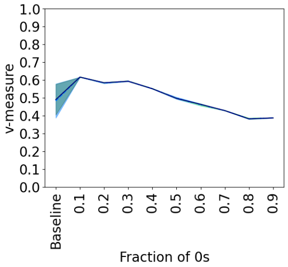

(b) S500_O

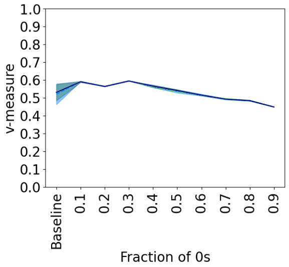

(c) S1000

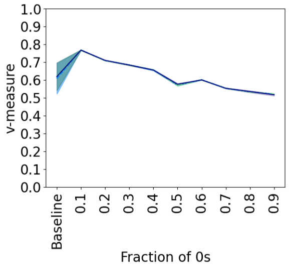

(d) S1000_O

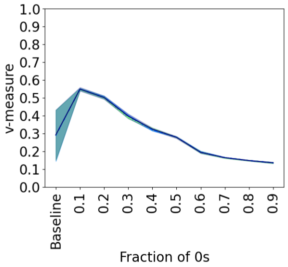

(e) S10000

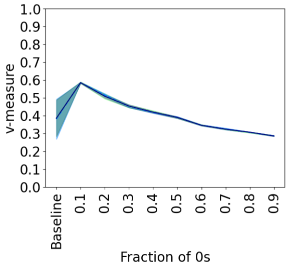

(f) S10000_O

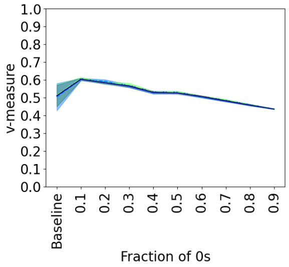

(g) S30000

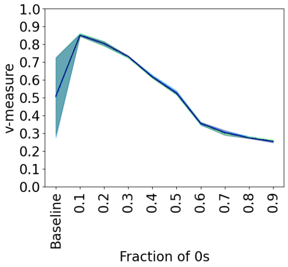

(h) S30000_O

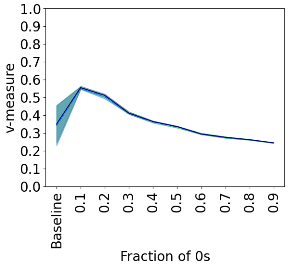

(i) S50000

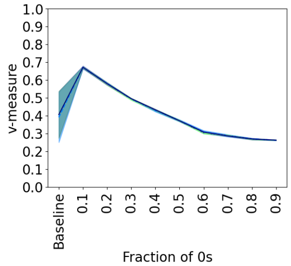

(j) S50000_O

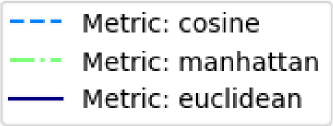

Figure 19: V-measure for an increasing number of dropouts against the baseline clustering for different Leiden metric parameters. Leiden clustering is performed with number of neighbors = 5 and number of dimensions = 2. Mean and standard deviation of 10 runs are indicated.

2.7 Imputation does not Improve Leiden Cluster Stability or Quality

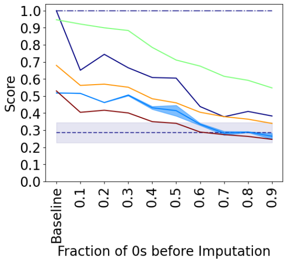

(a) S500_I

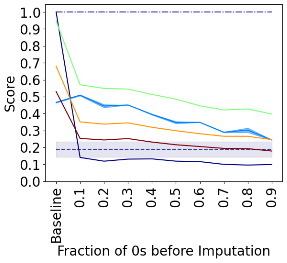

(b) S500_I_O

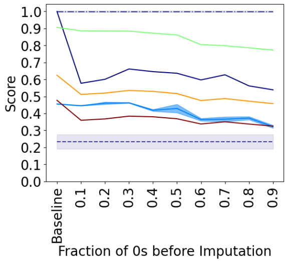

(c) S1000_I

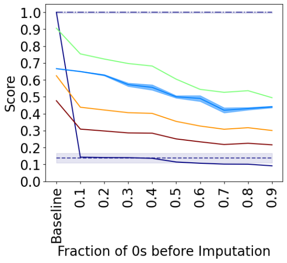

(d) S1000_I_O

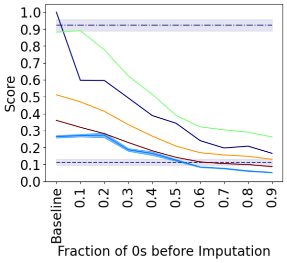

(e) S10000_I

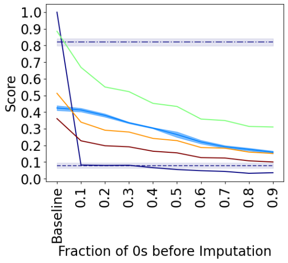

(f) S10000_I_O

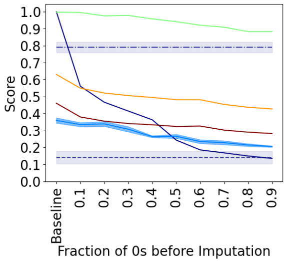

(g) S30000_I

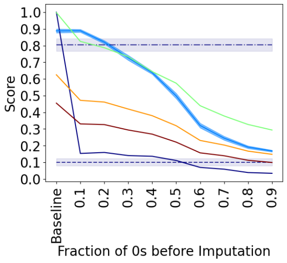

(h) S30000_I_O

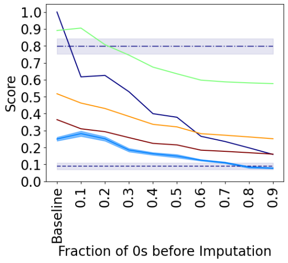

(i) S50000_I

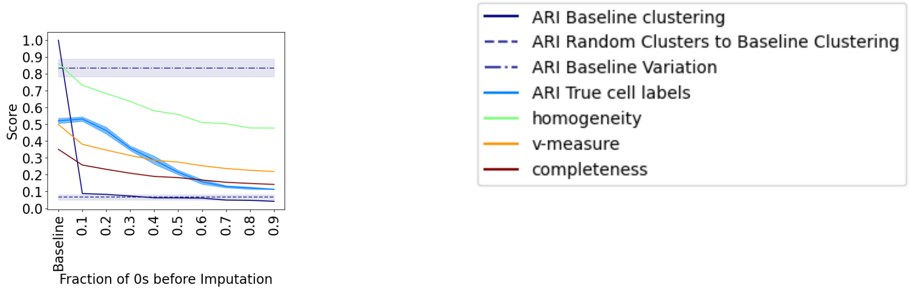

(j) S50000_I_O

Figure 20: ARI (dark blue) between Leiden clustering on the Baseline data in comparison to Leiden clustering on data with increasing fraction of 0s after imputation. The ARI towards the baseline clustering for random assigned clusters (−−) and for multiple runs on the baseline data (−.−) are indicated. In addition the ARI to the true class labels (light blue) are plotted for multiple runs, where mean and standard-deviation are indicated. Cluster quality is computed by homogeneity (green), completeness (red) and the v-measure (yellow).

References

[1] Irene Papatheodorou, Pablo Moreno, Jonathan Manning, Alfonso Muñoz-Pomer Fuentes, Nancy George, Silvie Fexova, Nuno A Fonseca, Anja Füllgrabe, Matthew Green, Ni Huang, et al. Expression atlas update: from tissues to single cells. *Nucleic acids research*, 48(D1):D77–D83, 2020.

[2] Nancy George, Silvie Fexova, Alfonso Munoz Fuentes, Pedro Madrigal, Yalan Bi, Haider Iqbal, Upendra Kumbham, Nadja Francesca Nolte, Lingyun Zhao, Anil S Thanki, et al. Expression atlas update: insights from sequencing data at both bulk and single cell level. *Nucleic Acids Research*, 52(D1):D107–D114, 2024.

[3] Xiuwei Zhang, Chenling Xu, and Nir Yosef. Simulating multiple faceted variability in single cell rna sequencing. *Nature communications*, 10(1):2611, 2019.
